# Learning about the X from our parents

**DOI:** 10.3389/fgene.2015.00015

**Published:** 2015-02-10

**Authors:** Alison S. Wise, Min Shi, Clarice R. Weinberg

**Affiliations:** ^1^Biostatistics and Computational Biology Branch, National Institute of Environmental Health Sciences, Research Triangle ParkDurham, NC, USA; ^2^Department of Biostatistics, Gillings School of Global Public Health, University of North Carolina at Chapel HillChapel Hill, NC, USA

**Keywords:** X chromosome, family-based design, case-parent triad, oral cleft, association study, SNPs, likelihood ratio test

## Abstract

The X chromosome is generally understudied in association studies, in part because the analyst has had limited methodological options. For nuclear-family-based association studies, most current methods extend the transmission disequilibrium test (TDT) to the X chromosome. We present a new method to study association in case-parent triads: the parent-informed likelihood ratio test for the X chromosome (PIX-LRT). Our method enables estimation of relative risks and takes advantage of parental genotype information and the sex of the affected offspring to increase statistical power to detect an effect. Under a parental exchangeability assumption for the X, if case-parent triads are complete, the parents of affected offspring provide an independent replication sample for estimates based on transmission distortion to their affected offspring. For each offspring sex we combine the parent-level and the offspring-level information to form a likelihood ratio test statistic; we then combine the two to form a combined test statistic. Our method can estimate relative risks under different modes of inheritance or a more general co-dominant model. In triads with missing parental genotypes, the method accounts for missingness with the Expectation-Maximization algorithm. We calculate non-centrality parameters to assess the power gain and robustness of our method compared to alternative methods. We apply PIX-LRT to publically available data from an international consortium of genotyped families affected by the birth defect oral cleft and find a strong, internally-replicated signal for a SNP marker related to cleft lip with or without cleft palate.

## Introduction

The X chromosome is unique in that males have only one, maternally-derived copy, while females are diploid. As a form of dosage compensation, a random X is inactivated in each cell early in female embryonic development (Lyon, 2002). Regions on the X chromosome have been identified in association with several diseases, including Parkinson's disease (Nemeth et al., 1999; Scott et al., 2001; Pankratz et al., 2003) and autism (Shao et al., 2002; Vincent et al., 2005; Piton et al., 2011). However, the X lags behind its autosomal counterparts in association and linkage findings, in part due to the need to use methods specific for X-linked markers (Wise et al., 2013).

Most family-based methods available for X chromosome analysis are extensions of autosomal methods. The original transmission/disequilibrium test (TDT) was proposed to detect autosomal SNPs associated with disease in case-parent triads (Spielman et al., 1993). For studies that also include unaffected siblings and may or may not include parental genotyping, we have the sibling TDT (S-TDT) (Spielman and Ewens, 1998) and the reconstruction-combination TDT (RC-TDT) (Knapp, 1999). These family-based methods were extended to X-linked markers with the XTDT, XS-TDT, and XRC-TDT (Horvath et al., 2000). A number of extensions have been developed to accommodate larger families (Ding et al., 2006; Chung et al., 2007). A version of FBAT (Laird et al., 2000) can also be used for the X chromosome and generalizes the XTDT. These methods provide *p*-values to test for association; they do not enable estimation of disease-related marker relative risks. The method we will propose is for case-parent triads, but accommodates triads with a missing individual.

Likelihood-based log-linear multinomial modeling approaches for nuclear families can use the EM algorithm to handle missing autosomal SNP genotypes (or individuals), and provide both robustness against bias due to population stratification and the opportunity to estimate disease-related marker relative risks (Weinberg et al., 1998; Rampersaud et al., 2007). HAPLIN is a likelihood-based method that is able to estimate relative risks for single SNPs and haplotypes on the autosomes and X chromosome (Gjessing and Lie, 2006; Jugessur et al., 2012). However, as we are interested in methods that do not assume Hardy-Weinberg equilibrium (HWE), which HAPLIN requires, we will not discuss the method further.

The X-LRT, a log-linear likelihood ratio test of association for X-linked markers that does not assume HWE, was recently developed (Zhang et al., 2008). This method performs well, compared to transmission-based methods, and allows male and female offspring to have separate relative risks. The X-LRT conditions on parental mating type (the pair of parental genotypes), which we will show can cause bias because families with female and male affected offspring are forced to share the same mating type parameters. We present a new method, the sex-stratified X chromosome likelihood ratio test (SSX-LRT), which prevents that bias by allowing distinct mating type parameters for male vs. female affected offspring.

We show that additional improvement is possible by exploiting genotype information in the parents not used in previous methods. Mothers and fathers of affected offspring are differentially enriched for susceptibility markers depending on the sex of the affected offspring. We demonstrate that an assumption of parental allelic “exchangeability” enables the added information to be captured in a way that resists bias due to population stratification. Consequently, regardless of what alleles parents transmit to their affected offspring, additional information can be robustly gleaned from the parental X genotypes to supplement the transmission-based SSX-LRT, creating the “parent-informed X chromosome likelihood ratio test” (PIX-LRT).

In the following sections, we initially describe the SSX-LRT and PIX-LRT for single X-linked SNP markers with complete genotype data. An extension of the approach then enables inclusion of families with missing genotype data. We assess Type I error rates for SSX-LRT, PIX-LRT, and X-LRT and compare power for the SSX-LRT, PIX-LRT, and XTDT by calculating chi-squared non-centrality parameters based on expected counts (Agresti, 2012). As an example, we apply the PIX-LRT to family data from an oral cleft dataset to analyze SNP markers on the X chromosome. We conclude with a discussion of the advantages and limitations of PIX-LRT, and our SNP findings for cleft lip.

## Subjects and methods

### Case-parent design and assumptions

Consider a sample of case-parent triads who have all been genotyped at a di-allelic X locus. Let *M, F*, and *C* denote the number of copies of the variant (minor) allele in the mother, father and affected offspring (proband), respectively. We exclude regions on the X that correspond to a homologous region on Y, including the pseudo-autosomal regions and the X-transposed region (PARs, XTR). Then *M* ∈ {0, 1, 2}, *F* ∈ {0, 1}, *C* ∈ {0, 1} for male offspring, and *C* ∈ {0, 1, 2} for female offspring. Consider tests of the null hypothesis that there is no association or no linkage against the alternative of association in the presence of possible linkage. Assume there is Mendelian transmission at that locus in the source population. Further assume parental allelic exchangeability in the source population, as in (Shi et al., 2008); that is, within a mating pair, the variant alleles are randomly located across the three X chromosomes. This assumption, which is met under non-assortative mating within subpopulations at that locus, can be tested within the source population using the following model for the expected number of families, based on the sample size times the probabilities in Table 1:

(1) $$\begin{array}{l} \ln\left(E[N_{M,F}|M+F]\right)=\log(\mu_{M+F})+\alpha_{1}I_{\left(M\;=\;1,\;F\;=\;0\right)}\\ \;+\;\alpha_{2}I_{\left(M\;=\;1,\;F\;=\;1\right)}+\text{log}(2)\;I_{\left(M\;=\;1\right)}\;\;\;\;\; \end{array}$$

**Table 1:** **Probabilities of mating pairs conditional on mating sum when parental allelic exchangeability is present (exch), and when it is not (no exch)**.

**M + F**	**M**	**F**	**Pr (M, F|M + F, exch)**	**Pr (M,F|M + F, no exch)**
0	0	0	1	1
1	1	0	2/3	2exp(α_1_)/(1 + 2exp(α_1_))
	0	1	1/3	1/(1 + 2exp(α_1_))
2	2	0	1/3	1/(1 + 2exp(α_2_))
	1	1	2/3	2exp(α_2_)/(1+2exp(α_2_))
3	2	1	1	1

Here *E* denotes expected value and *N_M, F_* is the random multinomial count variable denoting the number of triads where the mother and father carry *M* and *F* copies of the variant allele, respectively. μ_*M* + *F*_ are nuisance parameters that stratify families by conditioning on the sum of parental genotypes and log(2) is an offset term required because there are two ways for *M* to equal 1 (the variant allele can be on either chromosome). The parameters α_1_ and α_2_ are the log of half the odds that the mother carries 1 copy of the variant when the parents together have 1 and 2 copies, respectively. See Table 1. We can calculate a likelihood ratio test statistic for α_1_ = α_2_ = 0, which under exchangeability is distributed as a central chi-squared with two degrees-of-freedom (see Supplement S.1 for closed-form solutions). Note that parental allelic exchangeability is much less restrictive than assuming Hardy-Weinberg equilibrium (HWE) because it must hold only within unknown genetic subpopulations. Lastly, as is generally required for family studies we assume that variants are not determinants of fetal survival or parental ability to reproduce.

### Modification of the X-LRT to achieve robustness

The X-LRT (Zhang et al., 2008) provides a powerful likelihood ratio test for triad data with affected sons and daughters and also allows one to estimate disease-related marker relative risks. A multinomial likelihood is expressed in terms of offspring genotype relative risks. X-LRT conditions on the parental mating type by including mating type parameters, but forces those parameters to be the same for families with affected male and female offspring. That approach can consequently be biased (shown in results) when subpopulations have different minor allele frequencies (MAFs) and disease risks in males vs. females (non-carriers) differ among subpopulations. This bias can also occur when recruitment rates for families with male vs. female affected offspring differ across subpopulations with different minor allele frequencies. To remove this bias we stratify by both the parental mating type and the sex of the affected offspring (see Table 2). Let *aff* denote the event that the offspring is affected and define the relative risks, within parental mating type, as follows:

$$\begin{array}{l} e^{\beta_{1}}=R_{G1}\text{​}=\text{​}\mathrm{Pr}\left(\text{aff }|\text{ girl},C=1\right)/\mathrm{Pr}\left(\text{aff }|\text{ girl},C=0\right)\\ e^{\beta_{2}}=R_{G2}=R_{G1}\mathrm{Pr}\left(\text{aff }|\text{ girl},C=2\right)/\mathrm{Pr}\left(\text{aff }|\text{ girl},C=1\right)\\ e^{\beta_{3}}=R_{B}\text{​}=\text{​}\mathrm{Pr}\left(\text{aff }|\text{ boy},C=1\right)/\mathrm{Pr}\left(\text{aff }|\text{ boy},C=0\right) \end{array}$$

**Table 2:** **For affected sons and daughters, case-parent genotype probabilities using transmission information**.

		**Affected Son**			**Affected Daughter**		
**M**	**F**	**C**	**Pr(C|M,F,b)**	**E(*N_M,F,b_*)**	**C**	**Pr(C|M,F,g)**	**E(*N_M,F,g_*)**
0	0	0	1	γ_00*b*_	0	1	γ_00*g*_
0	1	0	1	γ_01*b*_	1	1	γ_01*g*_
1	0	0	1/(1 + *R_B_*)	γ_10*b*_	0	1/(1 + *R*_*G*1_)	γ_10*g*_
		1	*R_B_*/(1 + *R_B_*)		1	*R*_*G*1_/(1 + *R*_*G*1_)	
1	1	0	1/(1 + *R_B_*)	γ_11*b*_	1	*R*_*G*1_/(*R*_*G*1_ + *R*_*G*2_)	γ_11*g*_
		1	*R_B_*/(1 + *R_B_*)		2	*R*_*G*2_/(*R*_*G*1_ + *R*_*G*2_)	
2	0	1	1	γ_20*b*_	1	1	γ_20*g*_
2	1	1	1	γ_21*b*_	2	1	γ_21*g*_

The analysis follows a multinomial for the counts based on both triad genotypes and sex (*g* for girl and *b* for boy) of the affected offspring (*N_M,F,C,sex_*), modeled in a log-linear form, multiplying the sex-specific expected counts for each parental genotype pair by the probabilities shown in Table 2, as follows:

(2) $$\begin{array}{l} \ln\left(E\left[N_{M,F,C,sex}\right]\right)=log(\gamma_{M,F,sex})+\beta_{1}I_{\left(C\;=\;1,\;sex\;=\;g\right)}\\ \;+\;\beta_{2}I_{\left(C\;=\;2,\;sex\;=\;g\right)}+\beta_{3}I_{\left(C\;=\;1,\;sex\;=\;b\right)}\;\;\;\;\;\; \end{array}$$

Here γ_*M,F,sex*_ are 12 nuisance parameters that serve to confer robustness against population stratification by stratifying families by both mating type and sex of affected offspring. Exponentiating the β's produces the relative risk estimates (e.g., $e^{{\hat{\beta }}_{1}}={\hat{R}}_{G1}$). Inclusion of three unconstrained relative risk parameters allows one to avoid imposing an arbitrary relationship between the relative risks in boys and girls. We therefore refer to this method as the sex-stratified X-LRT (SSX-LRT). The corresponding log-likelihood for each sex (using the lower case “*n*” to denote observed counts of the variable *N*) is proportional to:

(3) $$\sum_{M,F,C}n_{M,F,C,sex}\log\left(\mathrm{Pr}\left(M,F,C\;|\text{ aff},\text{ sex}\right)\right)$$

Expression 3 can be rewritten as:

(4) $$\begin{array}{l} \sum_{M,F,C}n_{M,F,C,sex}\log\text{​​​}(\mathrm{Pr}\left(C\;|\;M,F,\text{aff},\text{sex}\right)\;\\ \;*\mathrm{Pr}\left(M,F\;|\text{ aff},\text{ sex}\right)) \end{array}$$

With complete data, the nuisance parameters do not need to be explicitly estimated when calculating the maximum likelihood estimates for the relative risks and the likelihood ratio test statistic. Closed form solutions to the maximum likelihood equations for the relative risks and the corresponding likelihood ratio test statistic under specified genetic models of inheritance are given in Supplement S.2. Note that triads with affected sons do not need genotyped fathers for the SSX-LRT.

When some genotype information is missing, we use the Expectation-Maximization (EM) algorithm as has been described (Weinberg, 1999). For the EM, the mating type parameters are needed to calculate the maximum likelihood estimators of the relative risks and the likelihood ratio test statistic. If two subpopulations have different minor allele frequencies and also different degrees of missingness, the missingness can be informative and use of the EM can induce bias in the estimate. To avoid this bias, if the subpopulations are identifiable (e.g., the analyst can stratify on ancestry) the EM can be run on a likelihood that allows different mating type parameters for each.

To form a test based on both families of affected sons and families of affected daughters, we recommend forming a combined test statistic. Let *X_B_* and *X_G_* be the one degree-of-freedom LRT chi-squared statistics based on families with affected sons and daughters, respectively, where *X_G_* is based on the coding: *R*^2^_*G*1_ = *R*_*G*2_. Under the null the sum of the two (independent) test statistics has a chi-squared distribution with two degrees-of-freedom.

However, rather than just computing the sum we note that the most plausible departures from the null would involve scenarios where the boys and girls experience the same direction of effect, that is, the variant either increases risk for both or decreases risk for both. (In fact, because the two test statistics are statistically independent, one could regard families with affected daughters as a replication sample for findings based on families with affected sons.) Accordingly, the following construction exploits that directional agreement to enhance power (see Zaykin, 2011) for a combined test. Take the square root of each chi-squared statistic and attach to that square root the sign corresponding to the direction of the estimated effect, *S_B_* and *S_G_* (“+1” for relative risk >1 and “-1” for relative risk <1). Under the combined null hypothesis the results will be two independent standard Gaussian statistics. Let *N_B_* and *N_G_* be the number of triads with a heterozygous mother and an affected son or daughter, respectively. The weighted combined Z statistic is constructed as follows:

(5) $$Z_{C}=\frac{S_{B}\sqrt{N_{B}X_{B}}+S_{G}\sqrt{N_{G}X_{G}}}{\sqrt{N_{B}+N_{G}}}\;\sim \;N\left(0,1\right)$$

*Z*^2^_*C*_ follows a central chi-squared distribution with one degree of freedom under the null and a non-central chi-squared under alternatives, where the non-centrality parameter is:

$${\left[E\left\{\frac{S_{B}\sqrt{N_{B}X_{B}}+S_{G}\sqrt{N_{G}X_{G}}}{\sqrt{N_{B}+N_{G}}}\right\}\right]}^{2}$$

Intuitively, if the number of informative families with an affected son is markedly different from the number with an affected daughter, this weighting scheme will favor the larger test statistic and sample size.

We focus on a sex-stratified analysis, but one could alternatively impose a relationship between *R_B_* and *R*_*G*1_, *R*_*G*2_. Such a model can be fitted by use of widely available software (e.g., glm in R Development Core Team, 2013) to maximize the multinomial likelihood and to estimate parameters. For example, under a simple model based on X-inactivation, one could argue for a two degree-of-freedom test with:

$$\begin{array}{l} H_{0}:R_{G1}=R_{G2}=R_{B}=1\;\left(\beta_{1}=\beta_{2}=\beta_{3}=0\right)\\ H_{a}:R_{G2}=R_{B}\neq 1\text{ or }R_{G1}\neq 1\;(\beta_{2}=\beta_{3}\neq 0\text{ or }\beta_{1}\neq 0) \end{array}$$

If we additionally assume a log-additive model in girls, we would have a one degree-of-freedom test with:

$$H_{a}:R_{G1}^{2}=R_{G2}=R_{B}\neq 1\;(2\beta_{1}=\beta_{2}=\beta_{3}\neq 0)$$

The log-linear form of the model would be:

$$\ln\left(E\left[N_{M,F,C,sex}\right]\right)=\log(\gamma_{M,F,sex})+\beta_{1}C\left[I_{\left(sex\;=\;g\right)}+2I_{\left(sex\;=\;b\right)}\right]$$

Similar analyses that either simplify the parameterizations or are aimed at testing X-inactivation relationships (where *R*_*G*2_ = *R_B_* is the null hypothesis to be tested) can also be carried out in the context of the PIX-LRT method to be described.

### PIX-LRT statistic

The likelihood we will maximize is based on two separate factors, one that models transmissions conditional jointly on both the sex of the affected offspring and the parental genotypes (cf. the stratum parameters in Table 2), i.e., *M*, *F* (as described above for SSX-LRT), and another that models *M*, *F* conditional on *M+F* and the sex of the affected offspring (cf. Table 1). The second, parental-information component is statistically independent of the transmission-based component, allowing parental data to provide a kind of internal replication. That parental piece has not been explicitly exploited by other methods.

The transmission-based part of the information is very much like that captured by the SSX-LRT just described in the Section Modification of the X-LRT to Achieve Robustness, through maximizing expression (4) above. But PIX-LRT will augment that by capturing information from the parents, rather than conditioning away that information (cf. the 12 stratification parameters included in Table 2), by instead conditioning more coarsely on the total number of copies of the variant carried by the two parents.

We begin with some intuition to clarify why there is information in how a fixed number of variant alleles (*M* + *F*) is distributed across the two parents. Under the null hypothesis, for a SNP on the X chromosome, one would expect neither the mother's two chromosomes nor the father's single chromosome to be enriched for either allele. However, suppose the variant is linked to risk of the disease. Because the mother of an affected son was the source of his only X, the mothers of affected sons should be enriched for that variant as compared to the fathers. Because a father of an affected daughter transmitted his only X to his daughter, whereas the mother could transmit either one of her two X's to her daughter, the fathers of affected daughters should be enriched compared to the mothers. These resulting opposing patterns of enrichment within the parents can be exploited by conditioning on the sex of the affected offspring and the number of variant alleles the parents carry (*M* + *F*), taking advantage of our parental exchangeability assumption.

Specifically, for each sex, one can augment the earlier analysis by incorporating the following log likelihood to capture the parent-only information:

(6) $$\sum_{M,F}n_{M,F,sex}\log\left(\mathrm{Pr}\left(M,F|M+F,\text{aff},\text{sex}\right)*\mathrm{Pr}\left(M+F|\text{aff},\text{sex}\right)\right)$$

The probabilities used in expression 6 are given in Table 3. For complete data, closed-form maximum likelihood estimates of the relative risk and a likelihood ratio test statistic could be obtained from this method ignoring the genotype of the affected offspring and instead using only parents (see Supplement S.3). The EM can be used when genotype data are missing.

**Table 3:** **Relative risks and mating type probabilities associated with parental sum given affected offspring**.

				**Affected Sons**	**Affected Daughters**
***M* + *F***	***M***	***F***	**Null Prob**	**Pr(M, F|M + F)**	**Pr(M, F|M + F)**
0	0	0	1	1	1
1	1	0	2/3	(1 + *R_B_*)/(2 + *R_B_*)	(1 + *R*_*G*1_)/(1 + 2*R*_*G*1_)
	0	1	1/3	1/(2 + *_B_*)	*R*_*G*1_/(1 + 2*R*_*G*1_)
2	2	0	1/3	*R_B_*/(1 + 2*R_B_*)	*R*_*G*1_/(2*R*_*G*1_ + *R*_*G*1_)
	1	1	2/3	(1 + *R_B_*)/(1 + 2*R_B_*)	(*R*_*G*1_ + *R*_*G*2_)/(2*R*_*G*1_ + *R*_*G*2_)
3	2	1	1	1	1

The combined likelihood that now includes both the parental data and the transmission data can be written as a multinomial (see Table 4) and modeled in a log-linear form as follows:

(7) $$\begin{array}{l} \ln\left(E\left[N_{M,F,C,sex}|M+F\right]\right)=\;\\ \;\log(\mu_{M\;+\;F,sex})\;+\text{​​}\beta_{1}I_{\left(C\;=\;1,\;sex\;=\;g\right)}\\ \;+\beta_{2}I_{\left(C\;=\;2,\;sex\;=\;g\right)}\text{​​}+\;\beta_{3}I_{\left(C\;=\;1,\;sex\;=\;b\right)} \end{array}$$

**Table 4:** **For affected sons and daughters, case-parents genotype probabilities using parental sum information**.

			**Affected Sons**			**Affected Daughters**		
**M + F**	**M**	**F**	**C**	**Pr(M,F,C|M + F)**	***E*(*N_M_* + *F*)**	**C**	**Pr(M,F,C|M + F)**	***E*(*N*_M_ + *F*)**
0	0	0	0	1	μ_0*b*_	0	1	μ_0*g*_
1	0	1	0	1/(2 + *R_B_*)	μ_1*b*_	1	*R*_*G*1_/(1 + 2*R*_*G*1_)	μ_1*g*_
	1	0	0	1/(2 + *R_B_*)		1	*R*_*G*1_/(1 + 2*R*_*G*1_)	
	1	0	1	*R_B_*/(2 + *R_B_*)		0	1/(1 + 2*R*_*G*1_)	
2	1	1	0	1/(1 + 2*R_B_*)	μ_2*b*_	2	*R*_*G*2_/(2*R*_*G*1_ + *R*_*G*2_)	μ_2*g*_
	1	1	1	*R_B_*/(1 + 2*R_B_*)		1	*R*_*G*1_/(2*R*_*G*1_ + *R*_*G*2_)	
	2	0	1	*R_B_*/(1 + 2*R_B_*)		1	*R*_*G*1_/(2*R*_*G*1_ + *R*_*G*2_)	
3	2	1	2	1	μ_3*b*_	2	1	μ_3*g*_

As before, inclusion of three unconstrained relative risk parameters allows one to avoid imposing an arbitrary relationship on the relative risks in boys and in girls. The corresponding likelihood for PIX-LRT for each sex is then proportional to:

(8) $$\begin{array}{l} \sum_{M,F,C}n_{M,F,C,sex}\log(\mathrm{Pr}\left(M,F,C\;|\;M+F,\text{aff},\text{sex}\right)\\ \;*\;\mathrm{Pr}\left(M+F\;|\text{ aff},\text{sex}\right)) \end{array}$$

For complete data, closed-form solutions to the maximum likelihood equations for the relative risks and the corresponding likelihood ratio test statistic are given in Supplement S.4. The number of informative families is greater for PIX-LRT than SSX-LRT; families where *M* = 0, *F* = 1 and *M* = 2, *F* = 0 are informative for PIX-LRT but not for SSX-LRT. The partial information can be used for all triads where at least one member has genotype data. A combined score can be calculated for the PIX-LRT as was described for SSX-LRT. However, *N_B_* and *N_G_* are now the number of informative families, that is, families for which *M* + *F* cannot be inferred to be 0 or 3 with an affected son or daughter. This method is available as an R package at http://www.niehs.nih.gov/research/resources/software/biostatistics/pixlrt/index.cfm.

### Type I error rate and power calculations

The Type I error rate and the power are assessed by calculating the non-centrality parameter (NCP) for the distribution of a chi-squared likelihood ratio test statistic. Under the null hypothesis, the LRT statistic follows a central chi-squared distribution, which has an NCP of 0. The NCP is calculated by treating expected triad counts under the specified population structure as data used to fit the relevant models (O'brien, 1986; Agresti, 2012). Values of non-centrality parameters can be translated to power values using the non-central chi-squared distribution with the appropriate degrees of freedom.

To assess performance when there is admixture present in the population, we calculated the NCP for PIX-LRT, SSX-LRT, XTDT, and X-LRT. Consider two scenarios, each with two subpopulations of equal size, with no effect of the variant allele in either sex. In the first scenario, one subpopulation has a minor allele frequency of 0.3, a disease risk of 0.02 in males, and 0.02 in females. The second subpopulation has a minor allele frequency of 0.2, a risk of 0.01 in males, and of 0.02 in females. A second scenario is similar except in the first subpopulation the disease risk is 0.03 in females and the second subpopulation has a disease risk of 0.02 in males. For computational convenience we assume HWE within each subpopulation. The expected counts were calculated for 1000 families with affected offspring. Non-centrality parameters were estimated for tests of (1) *H*_0_: no effect in males or females; (2) *H*_0*m*_: no effect in males; (3) *H*_0*f*_: no effect in females.

We compare PIX-LRT to X-LRT for a scenario where both are valid. We consider a setting in which there are 1000 triads, *R_B_* is 1.5, and *R*^2^_*G*1_ = *R*_*G*2_ = 2. In the non-carriers, the disease risk in boys is twice that in girls. We calculate power (based on non-centrality parameters) as a function of minor allele frequencies. We choose an alpha level of 5 × 10^−6^ as this approximates the alpha 0.05 Bonferroni-corrected value needed for the X chromosome. We modify X-LRT to account for a log-additive dose effect in girls. Therefore, the X-LRT test for a fetal effect involves two degrees-of-freedom.

We also compare power of the PIX-LRT to the SSX-LRT and XTDT, under a homogeneous population, for computational simplicity. To calculate the NCP for the XTDT we use the method proposed by Deng and Chen (2001). We do not include the other X chromosome extensions, as only complete triads are considered with no additional siblings or extended pedigrees.

For our power analysis we consider settings in which sex-specific tests are of interest to highlight similarities and differences between the two sexes. We consider the following 500-triad scenarios: affected male offspring and a minor allele frequency of either 0.3 or 0.1; affected female offspring, *R*^2^_*G*1_ = *R*_*G*2_, and a minor allele frequency of either 0.3 or 0.1. We plot the non-centrality parameters as a function of the relative risk (*R*_*G*1_ for girls), and include the corresponding power for a one-degree-of-freedom LRT at alpha level 5 × 10^−6^.

To study the EM algorithm in PIX-LRT and SSX-LRT, we use the same scenarios and set *R_B_* to 2, and *R*_*G*1_ to 2. We plot the non-centrality parameter (and the power at alpha level 5 × 10^−6^) as a function of the proportion of missing fathers, missing mothers, or a combination. For the combination scenarios, only one parent is missing, and twice as many fathers as mothers are assumed missing.

### Oral cleft data

We applied PIX-LRT with the EM to the X chromosome data from the International Consortium to Identify Genes and Interactions Controlling Oral Clefts. The data were downloaded from dbGaP (Mailman et al., 2007) (Accession number: phs000094.v1.p1, Beaty et al., 2010). The data were previously analyzed by Patel et al. (2013) using FBAT (Laird et al., 2000). Patel et al. (2013) used only complete triads and included all ethnicities in their joint analysis, whereas we included partial triads but only Asian (including Pacific Islanders) and Caucasian ethnicities. We analyzed 13,283 SNPs on the X chromosome that had a minor allele frequency in the parents greater than 0.02, and had a unique mapping from the Illumina Human610-Quad v1.0 Build 36 to Build 37. For a family-wise alpha of 0.05 with a Bonferroni correction, the cutoff for the *p*-value is 3.74 × 10^−6^.

We included all triads for which we have genotype data from the case, and the parents are not of differing ethnicity. Thirteen percent of the Asian triads and 21% of the Caucasian triads were incomplete. If multiple affected siblings were present, we randomly chose one sibling (27 siblings removed). We analyzed 1105 European families and 1286 Asian families. The clefting phenotype is divided into two categories: one is cleft palate only (denoted CPO) and the other is cleft lip with or without cleft palate (denoted CL/P). This phenotype split is based on genetic and embryological findings suggesting they are distinct (Murray, 2002). The gender and cleft subtype breakdown is shown in Table 5. Note that CL/P predominantly affects boys while CPO is slightly more common in girls.

**Table 5:** **Case-parent families by cleft type, gender and ancestry**.

	**European**		**Asian**		**Total**	
	**Male**	**Female**	**Male**	**Female**	**Male**	**Female**
**CLEFT TYPE**						
CL/P	539	296	675	353	1214	649
CPO	132	138	103	155	235	293
Total by gender	671	434	778	508	1449	942
Total	1105		1286		2391	

*CL/P is cleft lip with or without palate, CPO is cleft palate only*.

We first test to see if any SNPs violate parental allelic exchangeability with Equation 1, using all pairs of parents. We use a QQ plot of –log_10_(*p*-value) to look for violations in exchangeability. This allows an overall assessment of exchangeability, but we recognize that SNPs that are truly associated with oral cleft may tend to violate exchangeability.

We run PIX-LRT with the EM on Asian and Caucasian families together and separately, allowing for different mating type parameters for each ethnic category. When the analysis is on the individual populations, only SNPs with a MAF greater than 0.02 in each population are studied (11368 in Asians, 13156 in Caucasian). We test markers separately for cleft palate only (CPO) and cleft lip with or without palate (CL/P). The combined test statistic (1 df) is used to combine information from families with affected sons and daughters. For female triads, we applied a log-additive risk model (1 df). Plots of −log_10_(*p*-value) against the marker position along the X chromosome (as determined by Build 37) can identify regions of interest.

For CL/P, we compared our top five SNPs using PIX-LRT with the EM to the top five identified in Patel et al. (2013). For these SNPs, we apply SSX-LRT and the parent-only analysis (Equation 5) to complete triads stratifying on sex of the affected offspring to better understand similarities and differences between our two results. SSX-LRT and the parent-only analysis are independent when complete triads are used, which enables estimates of relative risks to be compared in terms of agreement for parental vs. offspring-based findings, and affected-boy families vs. affected-girl families.

## Results

### Non-centrality parameters

Under a null scenario, with an admixed population, where the relative risks are 1, the NCPs calculated for PIX-LRT and SSX-LRT (Equations 4 and 7) and XTDT are all zero, which ensures the nominal Type I error rate. Table 6 displays the NCP and Type I errors calculated for the X-LRT for an admixed population. The NCPs are all greater than 0, implying inflated Type I error rates.

**Table 6:** **Non-centrality parameter and corresponding Type I error rates for X-LRT**.

	**Scenario 1**	**Scenario 2**
	**X-LRT**	**X-LRT**
*H*_0_ : *R*_*G*1_ = *R*_*G*2_ = *R_B_* = 1	0.64 (0.09)	0.98 (0.11)
*H*_0*m*_ : *R_B_* = 1	0.10 (0.06)	0.13 (0.07)
*H*_0*f*_ : *R*_*G*1_ = *R*_*G*2_ = 1	0.38 (0.08)	0.62 (0.10)

*For 1000 triads for a null variant in an admixed population as calculated by X-LRT. Type I error rates for α = 0.05 are shown in parenthesis. Scenario 1: First subpopulation has a MAF of 0.3 and a disease risk of 0.02 in males and females. Second subpopulation has a MAF of 0.2 and a disease risk of 0.01 in males and 0.02 in females. Scenario 2: First subpopulation has a MAF of 0.3 and a disease risk of 0.02 in males and 0.03 females. Second population has a MAF of 0.2 and a disease risk of 0.03 in males and 0.02 in females. H_0_: no disease-locus effect in male or female, H_0m_: no effect in males and test done in boy-affected families, H_0f_: no effect in females and test done in girl-affected families*.

**Figure 1:**
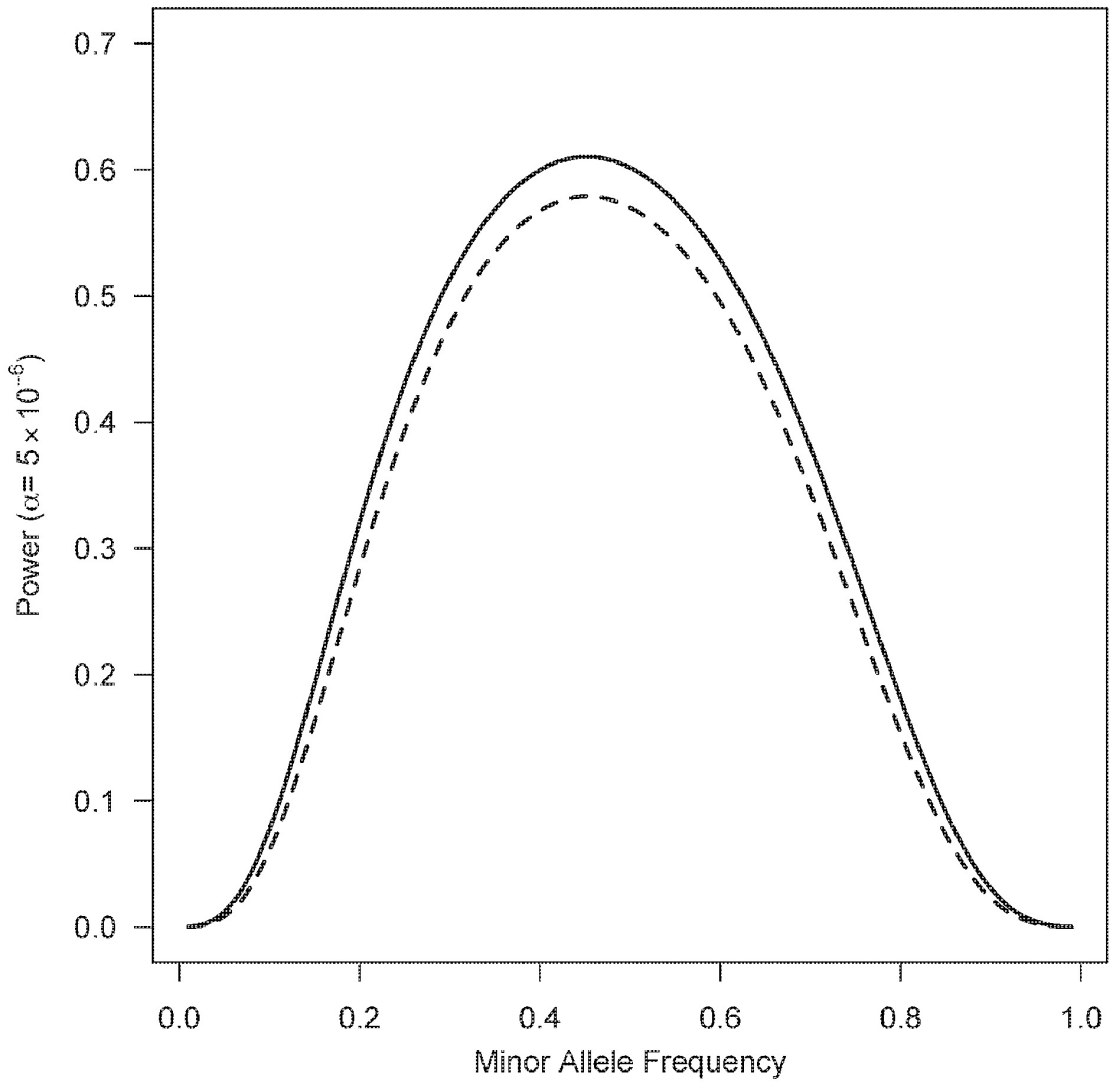
**Power estimates as a function of minor allele frequency of X-LRT and PIX-LRT**. Each analysis is based on 1000 triads with affected sons and daughters. *R_B_* = 1.5, *R*^2^_*G*1_ = *R*_*G*2_ = 2 and among non-carriers boys are twice as likely to have the disease as girls. Solid line represents PIX-LRT. Dashed line represents XLRT. PIX-LRT uses a one degree-of-freedom combined test, while X-LRT uses a two degree-of-freedom test.

**Figure 2:**
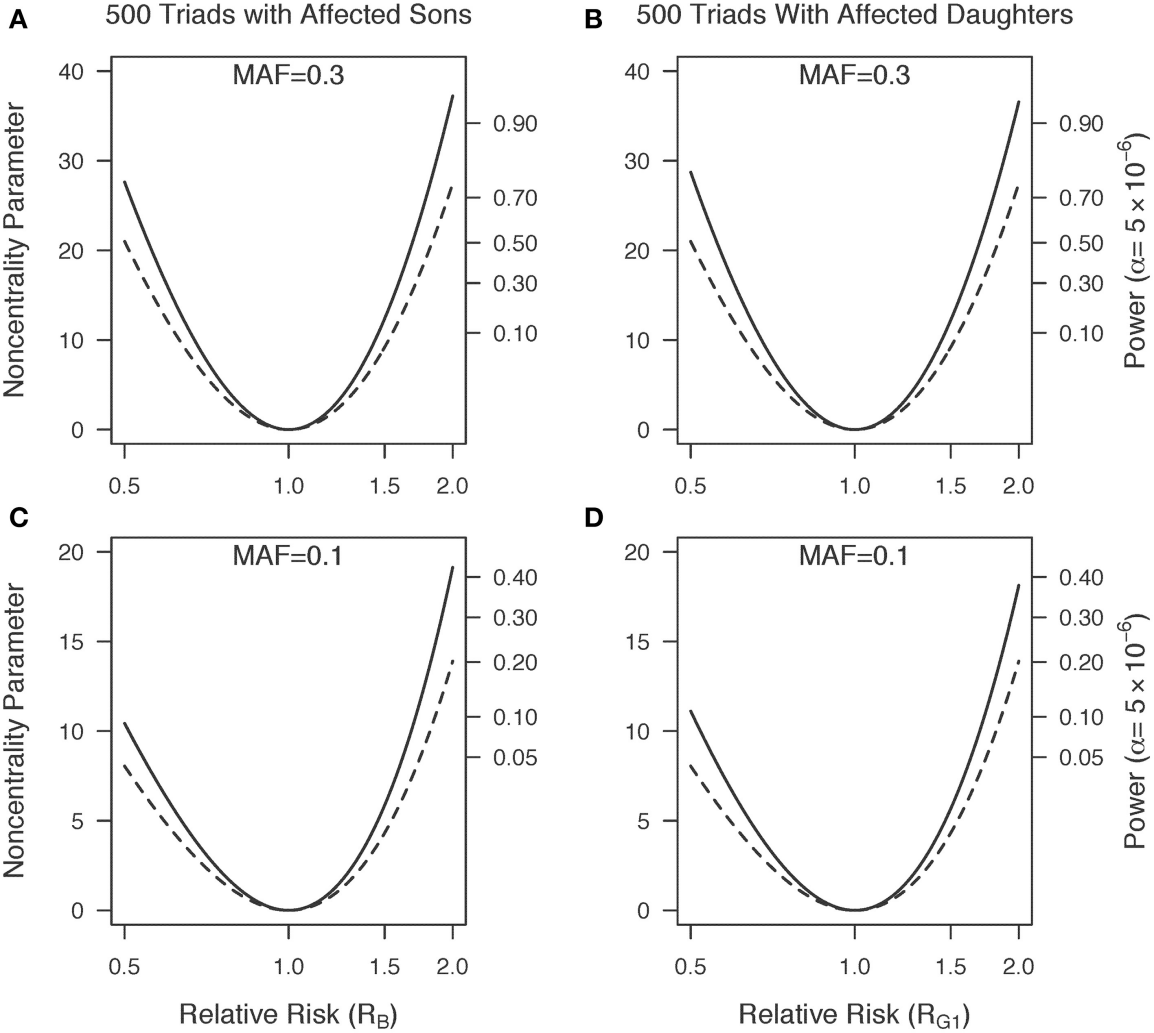
**Non-centrality parameter estimates as a function of relative risk. (A,C)** Five hundred triads with affected sons and **(B,D)** 500 triads with affected daughters. Minor allele frequencies of 0.3 **(A,B)** and 0.1 **(C,D)** are used. Solid lines represents PIX-LRT results. Dashed lines represents SSX-LRT and XTDT (plotted results were indistinguishable). PIX-LRT and SSX-LRT assume the relative risk in affected daughters is log additive in the number of copies of the minor allele.

Figure 1 shows a plot of the power for 1000 triads at a Type I error rate of 5 × 10^−6^ with a range of minor allele frequencies. The one degree-of-freedom combined PIX-LRT analysis outperforms the two degree-of-freedom X-LRT analysis. Figure 2 shows plots of the estimated NCP and the corresponding power for 500 triads at a Type I error rate of 5 × 10^−6^ with varying disease relative risks. For complete triads, PIX-LRT has higher NCPs (and corresponding power) than both the SSX-LRT and the XTDT. The SSX-LRT and XTDT perform similarly (see Discussion). For instance, for 500 triads with affected sons and a SNP with a minor allele frequency of 0.3 and relative risk of 2, the PIX-LRT has an estimated NCP of 37.22 (power = 0.94), the SSX-LRT has an estimated NCP of 27.45 (power = 0.75) and the X-TDT has an estimated NCP of 26.92 (power = 0.73). For this scenario, the expected number of informative triads used in PIX-LRT is 347.31 compared to 242.31 in SSX-LRT and XTDT. These estimates decrease if the minor allele frequency is 0.1.

The NCP plots for 500 triads with affected daughters are similar to those of affected sons (Figure 2, right compared to left). Under a log-additive model for girls, if the disease relative risk in sons equals the disease relative risk in heterozygous daughters ($R_{B}=R_{G1}=\sqrt{R_{G2}}$) then the estimated XTDT NCPs will be the same between the two sexes, and the estimated SSX-LRT NCPs will be the same (results not shown). The estimated PIX-LRT NCPs are close, but not identical. For *R*_*G*1_ = 2 and a MAF of 0.3, the estimated NCP is 36.57 (power = 0.93). If instead, the disease relative risk in sons equals the disease relative risk in daughters with two copies of the variant allele, (*R*_*B*1_ = *R*_*G*2_ = *R*^2^_*G*1_), then for $R_{G1}=\sqrt{2}$, the estimated PIX-LRT NCP is 8.86 (power = 0.06). Under this scenario, triads with affected sons offer greater power than those with affected daughters.

**Figure 3:**
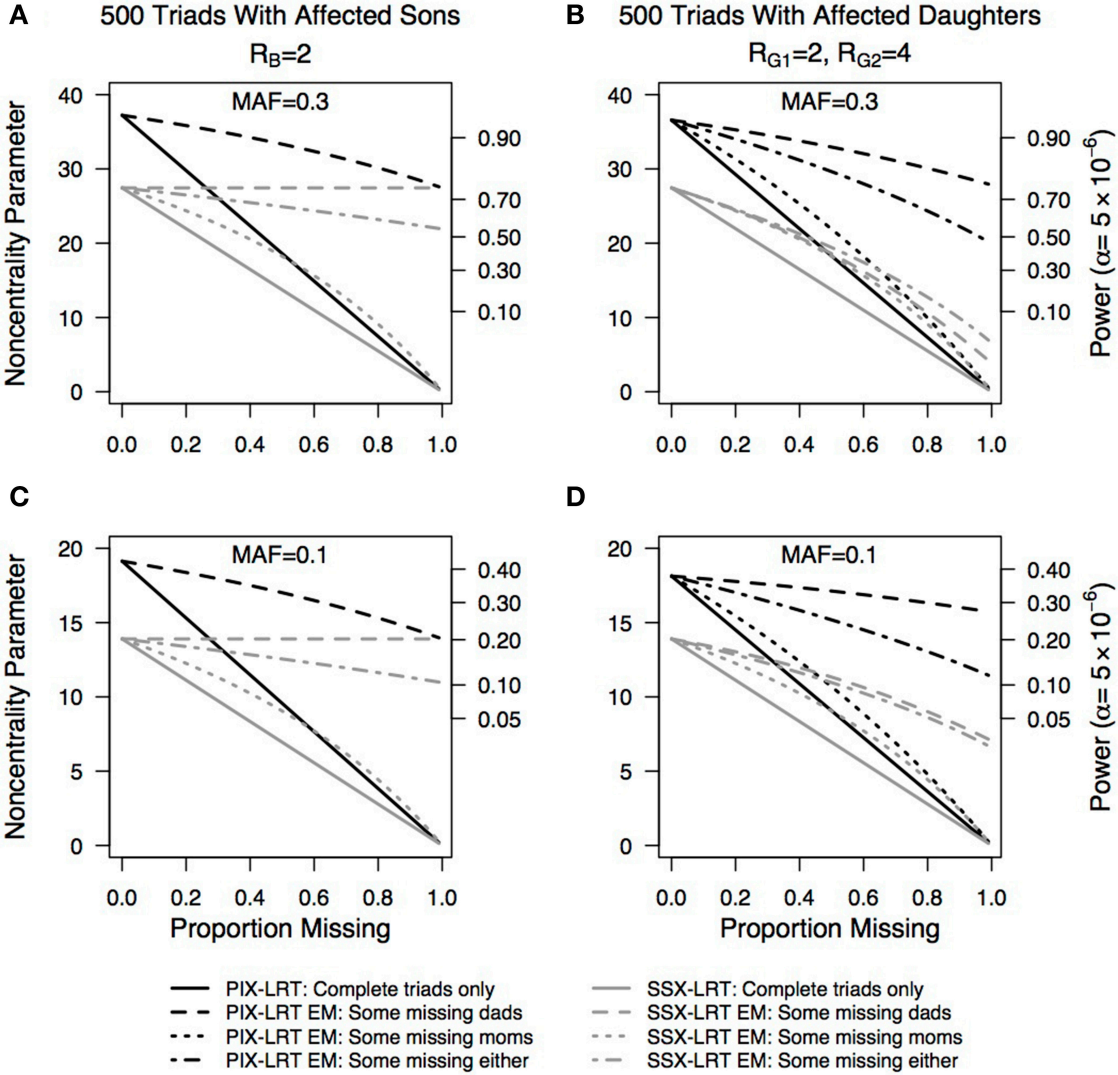
**Non-centrality parameter estimates as a function of missing parental genotypes using the Expectation-Maximization (EM) algorithm**. PIX-LRT and SSX-LRT were run on **(A,C)** 500 triads with affected sons and *R_B_* = 2 and **(B,D)** 500 triads with affected daughters, *R*_*G*1_ = 2 and *R*_*G*2_ = 4. Minor allele frequencies of 0.3 **(A,B)** and 0.1 **(C,D)** were used. Black lines represent PIX-LRT results. Gray lines represent SSX-LRT results. Solid lines represent results based on excluding incomplete triads (for sons, SSX-LRT can use triads with missing fathers). Long dashed lines represent results based on triads with the fathers missing (for PIX-LRT this single line represents either parent missing). Short/long dashed lines represent triads with either mother or father missing, with twice as many fathers missing than mothers. Short dashed lines represent results based on triads with mothers missing.

Plots of the effect of missing genotype data on the estimated NCP and the corresponding power at a Type I error rate of 5 × 10^−6^ are shown in Figure 3. Regardless of minor allele frequency, for triads with sons, PIX-LRT with the EM algorithm works equally well when some mothers are missing as when some fathers are missing (proof not shown). The SSX-LRT does not lose any power when fathers of sons are missing, as the fathers are non-informative. When mothers of sons are missing, we see the greatest power loss. In triads with daughters, regardless of minor allele frequency, more power can be recaptured from the EM when fathers are missing compared to mothers. This trend is seen in both PIX-LRT and SSX-LRT.

### Oral cleft

**Figure 4:**
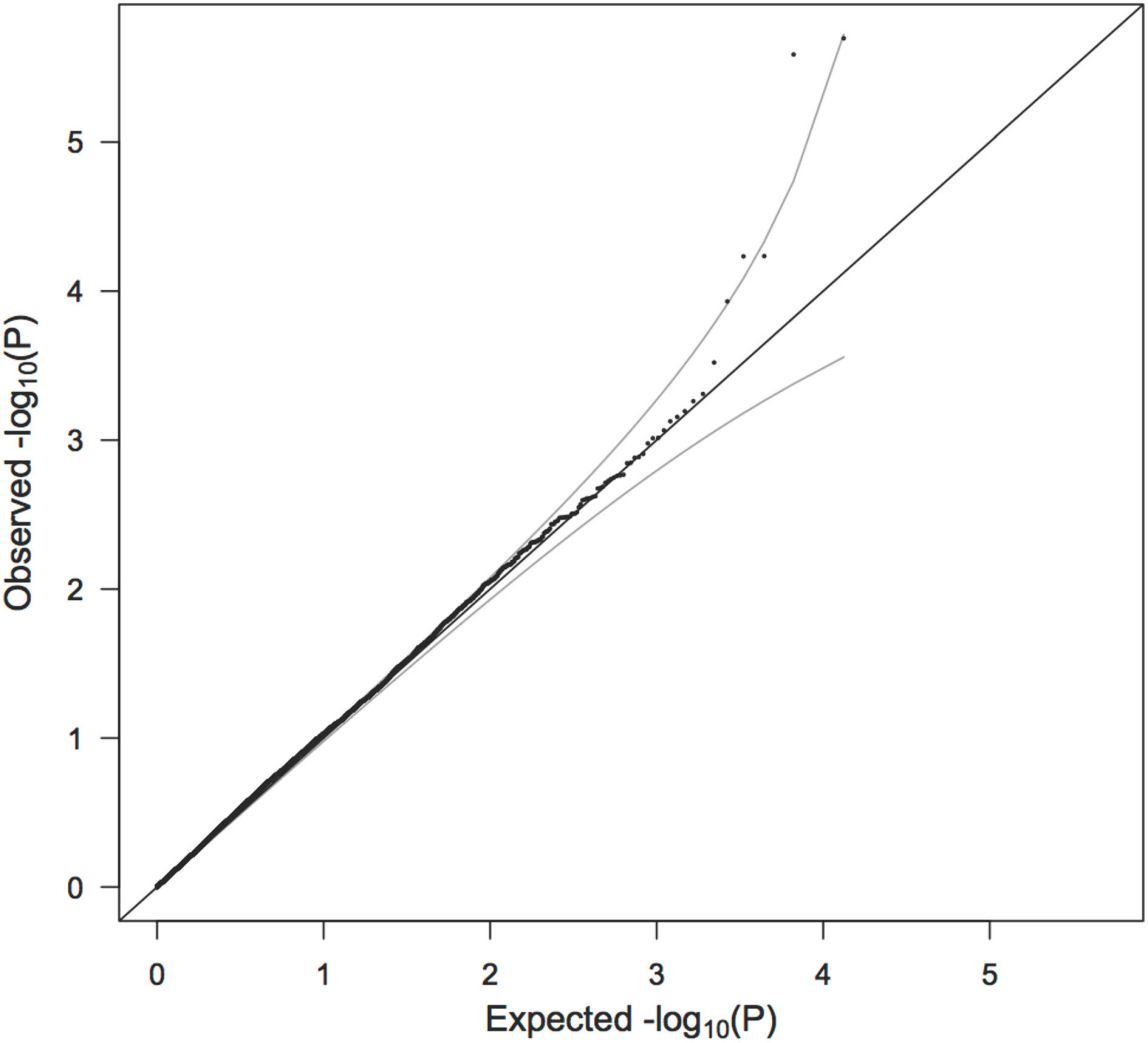
**QQ plot of −log_10_ (*p*) as calculated from the test of parental allelic exchangeability**. Ninety five percent confidence intervals are shown. Four SNPs (rs17269319, rs3747355, rs5906541, and rs12558269) are not shown because of extremely low *p*-values. No fathers carried the minor alleles for these four SNPs and the quality of genotyping consequently appears to be inadequate.

The QQ plot to assess parental exchangeability in the SNPs is shown in Figure 4. Four SNPs (rs17269319, rs3747355, rs5906541, and rs12558269) are not shown because their *p*-values (as calculated from Equation 1) are extreme outliers, less than 1 × 10^−16^. No father was found to carry any of these SNPs, despite some missing fathers having evidently transmitted the allele to their daughter. We consequently had reason to doubt the quality of the genotyping for those SNPs and omitted them from further analysis. [Patel et al. (2013) also noted that rs17269319 and rs12558269 had poor intensity plots.] The remaining points fell nicely on the QQ plot, except for 5 SNPs (rs2710404, rs5921330, rs1573667, rs7060927, and rs2266806) that raised concern about the parental exchangeability assumption. If these SNPs had appeared as top SNPs in the PIX-LRT analysis, those findings would need a closer look.

**Figure 5:**
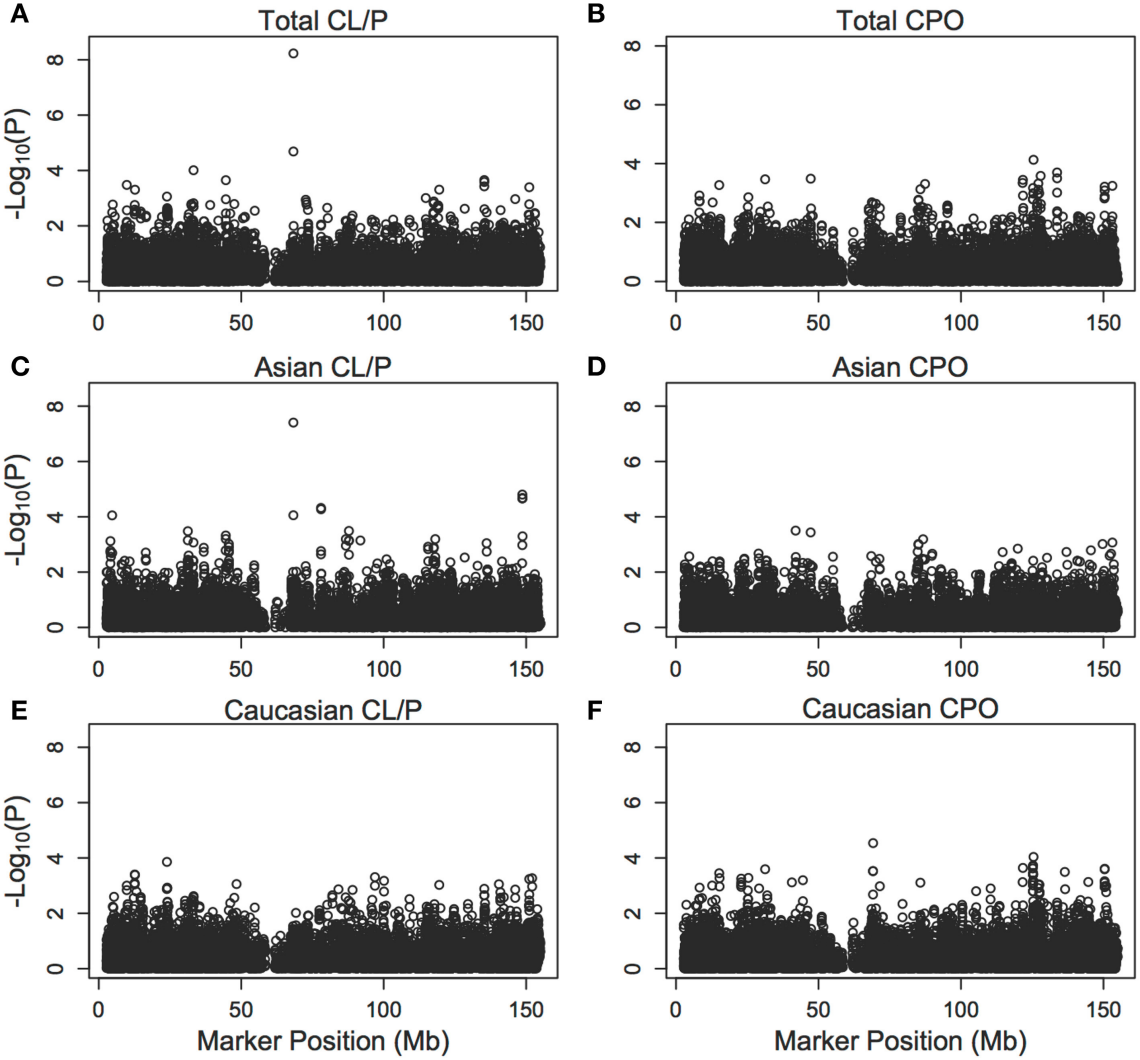
**Individual single nucleotide polymorphism significance of the cleft example**. The *p*-values [shown as −log_10_ (p)] are calculated from PIX-LRT with the EM using dbGaP data from families with oral cleft. A log-additive model is assumed for the risk in affected daughters and a combined score is used to combine the sex-specific statistics. Models were run on cleft lip with or without cleft palate families amongst **(A)** Asians and Caucasians, **(C)** Asians only, **(E)** Caucasians only, as well as cleft palate only families amongst **(B)** Asian and Caucasians, **(D)** Asians only, **(F)** Caucasians only.

Figure 5 shows results of the PIX-LRT with EM analysis of the SNPs along the X chromosome for CL/P and CPO in Caucasians and Asians separately and combined. The CPO analysis did not produce results suggestive of a marker related to CPO and no SNPs had *p*-values below the Bonferroni-corrected 3.76 × 10^−6^.

In CL/P analyses, we identified one SNP with a strong signal, rs5981162, the minor allele being associated with a decreased risk of cleft lip with or without palate (uncorrected *p*-value = 5.88 × 10^−09^). The PIX-LRT estimated disease relative risk within the combined Asian and Caucasian populations was 0.48 for male offspring carrying the variant allele, and 0.56 for female offspring carrying one copy of the variant allele (0.32 for two copies), showing good concordance. Similar relative risks are estimated in separate analyses of the Asian and Caucasian populations (see Table 7). The evidence for an effect is particularly strong in the Asian population, which has a higher variant allele frequency, and hence more informative families than the Caucasian population. The effect estimates based on parents of girls and parents of boys were also in good agreement with the offspring-based estimates (see Table 8). By contrast the PIX-LRT analysis of rs5981162 with CPO shows no effect (see Table 7), suggesting phenotypic specificity. Additionally, the test for parental allelic exchangeability produced a *p*-value of 0.18 for rs5981162, suggesting no violation of the assumption.

**Table 7:** **PIX-LRT analysis results of SNP rs5981162, located in the intergenic region between *ENFB1* and *PJA1* at basepair 68318753**.

**Cleft**	**Population**	**MAF^b^**	**Inf. boy fams^a^**	**Inf. girl fams^a^**	***P*-value**	***R_B_***	***R*_1_*_G_***
CL/P	All	0.076	415	146	5.88 × 10^−09^	0.48	0.56
	Asian	0.126	284	121	3.94 × 10^−08^	0.49	0.54
	Caucasian	0.016	131	25	4.24 × 10^−02^	0.38	0.72
CPO	All	0.076	78	65	0.544	0.85	0.91
	Asian	0.126	43	51	0.469	0.87	0.83
	Caucasian	0.016	35	14	0.895	0.77	2.35

*PIX-LRT with the EM was run on Asian and Caucasians separately and together. A log-additive model was used for triads with affected daughters and the combined score was calculated with the results from the sex stratified analysis*.

a *The number of informative triads at the marker*.

b *The minor allele frequency calculated from the parents in the population, not stratified by cleft type*.

**Table 8:** **Top 5 CL/P SNPs from our PIX-LRT analysis and from Patel et al. (2013)**.

**Our Top 5**	**Patel Top 5**	**Marker**	**Position**	**Gene**	**MAF^b^**	**Method**	**Comb. Z stat**	***R_B_***	***R*_*G*1_**
1	1	rs5981162	68318753	EFNB1,	0.076	PIX EM	−5.82	0.48	0.56
				PJA1^a^		SSX	−4.79	0.48	0.58
						Parent only	−3.09	0.45	0.51
2	–	rs5980788	68315938	EFNB1,	0.039	PIX EM	−4.26	0.49	0.54
				PJA1^a^		SSX	−4.01	0.45	0.53
						Parent only	−1.38	0.84	0.45
3	2	rs5928207	33244129	DMD	0.357	PIX EM	−3.90	0.73	0.87
						SSX	−4.72	0.67	0.74
						Parent only	−0.02	0.90	1.22
4	–	rs5930900	135296409	MAP7D3	0.370	PIX EM	3.69	1.22	1.30
						SSX	2.24	1.13	1.28
						Parent only	1.71	1.40	1
5	–	rs5905410	44584855	FUNDC1,	0.356	PIX EM	−3.69	0.81	0.72
				DUSP21^a^		SSX	−3.39	0.81	0.67
						Parent only	−1.99	0.77	0.72
–	3	rs5928208	33253904	DMD	0.390	PIX EM	−3.09	0.76	0.92
						SSX	−4.60	0.65	0.75
						Parent only	1.34	1.07	1.57
–	4	rs6631759	33239353	DMD	0.289	PIX EM	−3.17	0.75	0.90
						SSX	−4.45	0.66	0.73
						Parent only	0.55	0.99	1.33
–	5	rs5971698	33245234	DMD	0.366	PIX EM	−3.08	0.76	0.93
						SSX	−4.33	0.65	0.83
						Parent only	1.02	1.09	1.32

*The top 5 from Patel et al. (2013), after excluding SNPs that raised genotyping concern. Parent informed X-LRT (PIX-LRT) with the EM, Sex stratified X-LRT (SSX-LRT) on complete data, and a parent only analysis on complete data are used to calculate relative risk for CL/P in the combined Asian and Caucasian families. Combined Z statistics are used as opposed to LRT statistics to show direction of effect and are calculated from the sex-stratified analysis, as mentioned in the methods section*.

a *This marker lies in the intergenic region between the genes shown*.

b *The minor allele frequency calculated from the parents in the population, not stratified by cleft type*.

**Figure 6:**
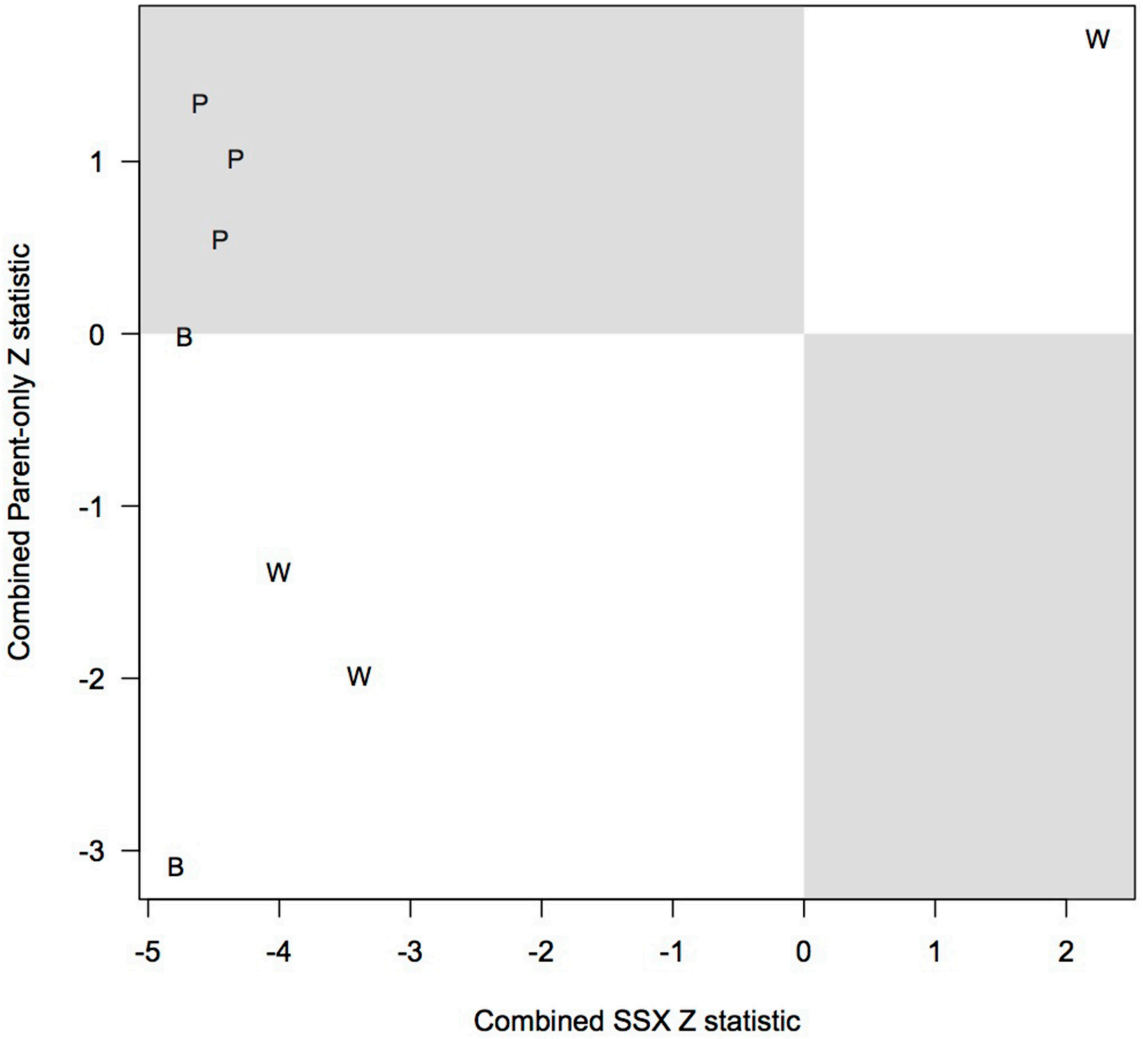
**Assessment of concordance through comparison of the parent-only Z scores and transmission (SSX) Z scores**. The figure shows the top five single nucleotide polymorphism (SNP) hits from the PIX-LRT analysis and Patel et al. (2013) in Asian and Caucasian families with cleft lip with or without palate. We excluded SNPs in the Patel et al. (2013) analysis that raised quality control concerns. The parent-only and SSX analyses assume the relative risk in affected daughters is log-additive in the number of copies of the variant allele. A combined score is used to combine the sex-specific statistics. “B” represents SNPs in the top 5 under both analyses, “P” represents SNPs that were in the top 5 for Patel et al. (2013) but not for us, “W” represents SNPs that were in the top 5 for our analysis but not Patel et al. (2013).

Table 8 compares the top 5 SNPs (based on the *p*-values) for CL/P within our analysis using PIX-LRT with EM and the top 5 SNPs based on the Patel et al. (2013) analysis. The top 5 SNPs from our analysis all showed no violation in the test of parental allelic exchangeability (the smallest *p*-value was 0.18). The SNPs rs17269319, rs5906541, and rs12558269 were excluded for quality control reasons, as discussed above (see Table 8 and Discussion). Two SNPs were in the top 5 under both analyses: rs5928207 and rs5981162 (our top hit). All triads for the 8 SNPs were analyzed with PIX-LRT with EM, and separate analyses using SSX-LRT and the parent only method, carried out to assess agreement, were based only on complete triads, to guarantee statistical independence. The combined Z-score (Equation 5) is shown in the table. Figure 6 plots the offspring-based SSX vs. the parent-only Z-score. SNPs that ranked high in the Patel analysis also have large SSX Z-scores. Evidence for a true hit is strengthened if the signs of the two independent statistics are in agreement, i.e., the points should ideally fall in the southwest or northeast quadrants of the figure. PIX-LRT identifies SNPs that have high offspring-based SSX and parent-only Z-scores in the same directions (i.e., concordance), the white-background quadrants in the plot. The PIX-LRT top hit, rs5981162, is in the southwest quadrant, showing strong evidence of a protective effect in both the offspring-based SSX and the parent-based analysis.

## Discussion

We have introduced new methods to analyze SNPs on the X chromosome: the SSX-LRT and the PIX-LRT. The SSX-LRT allows for stratification by sex of the affected offspring, which is based on the X-LRT but confers robustness against population stratification. The PIX-LRT then builds on the SSX-LRT by incorporating additional information in the parental genotypes that previous methods have not exploited. This information allows PIX-LRT to gain substantial power in identifying SNPs on the X chromosome associated with disease risk.

For situations in which both PIX-LRT and X-LRT are appropriate, under an assumed log-additive model for girls, the combined PIX-LRT outperforms X-LRT. The combined PIX-LRT enables a one degree-of-freedom test to be run. No assumption about the relationship between the male and female relative risks is made. Under this scenario, the X-LRT loses some power because it is a two degree-of-freedom test. For the X-LRT to be a one degree-of-freedom test, a relationship between the boy and girl relative risk must be asserted, and such a model may be mis-specified. It should be noted, however, that if the directions of the relative risks in boys and girls are opposite, then PIX-LRT loses power, while X-LRT does not.

As we showed in the results section, the parent-only portion of the PIX-LRT can also be used independently of the offspring-based transmission portion as a form of replication. This assessment of replication can only use complete triads however if the offspring-based and parent-based tests are to remain independent. The SNP that we identified as strongly protective based on PIX-LRT showed replication both across ethnic groups, across boys vs. girls and across parent-based results vs. offspring-based results, strengthening evidence for effect.

In general, with use of the EM algorithm more power is recaptured with missing fathers than with missing mothers (cf. Figure 3). This difference is driven by the daughter cases. For daughters, we can infer the father's genotype as long as the mother and daughter are not both heterozygous. However, with only the father's and daughter's genotypes, we cannot know the mother's genotype. For boys, in a transmission-based test (e.g., SSX-LRT), only the mothers are informative, so missing fathers do not affect the power of the test. However, in PIX-LRT, fathers are informative, and so when fathers of sons are missing, power is lost. For sons, when either parent is missing, the genotype of the complete triad cannot be known. For families with one parent and an affected son, the parents turn out to be equally informative (proof not shown).

While we demonstrated use of the EM algorithm for triads with a missing parent, there are circumstances where the genotype for the affected offspring might be missing. For example, in studying a defect such as anencephaly, following prenatal diagnosis a medically-indicated abortion might have been conducted. For families where only the parental genotype data is available, if the sex is known, the parent-only portion of the PIX-LRT can still be used in analyses of potential effects of variants on the X chromosome.

When, as in the oral cleft data used, families have missing parents, the EM enables use of their information. However, use of the EM can induce bias if a population has multiple subpopulations with both the minor allele frequencies and the extent of missingness varying across subpopulations. This bias is not specific to our method, and can be avoided via stratification if the subpopulations are identifiable.

For X-chromosome-wide association studies using case-parent triads, the power to detect an effect is influenced by the sex of the affected offspring. If the disease relative risk for a heterozygous female is less than that for a male carrier, as may be the case due to X-inactivation, the estimated power derived from the PIX-LRT, SSX-LRT, and X-TDT would typically be less for triads with daughters than for those with sons (Figure 2). Furthermore, for both SSX-LRT and PIX-LRT, missing mothers are at least as costly as missing fathers in their effects on power (Figure 3).

Some limitations deserve mention. The PIX-LRT estimates can be biased if an allele violates the parental exchangeability assumption, in which case the SSX-LRT may be a more appropriate method. In analyzing the oral cleft data we excluded the small fraction of differing-ethnicity parents, but including them did not noticeably affect the exchangeability QQ plot (data not shown). Transmission-based tests may also be biased if the violation is due to genotyping error or because the SNP is associated with fetal survival. If a SNP affects risk through a maternal effect (Wilcox et al., 1998), the parental contribution to the PIX-LRT results may be biased. Current research is extending the PIX-LRT to accommodate maternal effects.

Furthermore, the PIX-LRT and other X-chromosome methods are not suitable for the pseudo-autosomal regions (PAR) and the X-chromosome-transposed region (XTR). These regions have homologous regions on the Y chromosome, so that a male can have two copies of a SNP.

The NCP estimates obtained from SSX-LRT and the X-TDT are similar because these two tests are, respectively, the likelihood and score test for the same model. Schaid and Sommer (1994) showed that the TDT is the score test for a logistic regression allele dosage model (log additive). One can similarly show this for the XTDT.

We applied PIX-LRT to an international consortium of genotyped families affected by the birth defect oral cleft. In a previous analysis of the data, some of the most significant SNPs identified as by Patel et al. (2013) were not as significant when analyzed with PIX-LRT. An example is SNP rs5928208, which showed weaker results with PIX-LRT because the effect seen from the transmission analysis was not evident in the parent-only analysis. The top two SNPs in Patel, rs5906541 and rs17269319, and also rs3747355 and rs12558269, violated the mating exchangeability assumption. A harder look at the family genotypes was revealing in that their apparent absence in the fathers and the sons who were genotyped (as opposed to their inferred presence in fathers who were missing) raised concerns over the quality of genotyping for those SNPs.

With PIX-LRT, we identified rs5981162 as having a strong and protective effect on cleft lip with or with palate. This SNP was ranked fairly high in the previous analysis of the data by Patel et al. (2013), but PIX-LRT estimated sex-specific relative risks and found estimation concordance and a stronger *p*-value signal. The rs5981162 SNP is located between genes *EFNB1* and *PJA1*, and is downstream of these two genes. *EFNB1* is known to play a role in facial development: mutations on *EFNB1* are responsible for the majority of cases of craniofrontonasal syndrome (CFNS) (Twigg et al., 2004; Wieland et al., 2004), whose features can include cleft lip and palate. The SNP rs5981162 may potentially be located in a regulatory region of the *EFNB1* gene and functional studies could be illuminating.

### Conflict of interest statement

The authors declare that the research was conducted in the absence of any commercial or financial relationships that could be construed as a potential conflict of interest.

## Supplementary material

Supplementary Data **Supplemental data include four sections that present the closed form solutions and their statistical justifications**. Section 1 (S.1) presents how to test for the parental allelic exchangeability assumption. Section 2 (S.2) presents closed form solutions for the SSX-LRT. Section 3 (S.3) presents closed form solutions for the parent-only analysis. Section 4 (S.4) presents closed form solutions for PIX-LRT.
