# Supplementary material for: Learning about the X from our parents
Source: Front Genet. 2015 Feb 10;6:15. doi: 10.3389/fgene.2015.00015 (PMC4322752; doi:10.3389/fgene.2015.00015)
Supplement: Supplementary Data — Supplemental data include four sections that present the closed form solutions and their statistical justifications. Section 1 (S.1) presents how to test for the parental allelic exchangeability assumption. Section 2 (S.2) presents closed form solutions for the SSX-LRT. Section 3 (S.3) presents closed form solutions for the parent-only analysis. Section 4 (S.4) presents closed form solutions for PIX-LRT. [file DataSheet1.PDF]

S.1: Testing for the parental allelic exchangeability assumption

For the following, as in the main paper, we define *M* and *F* as the number of variant alleles carried by the mother and father. To test for parental allelic exchangeability define:

$$Q_1 = \Pr(M = 1|\mathrm{M} + \mathrm{F} = 1)$$
$$Q_2 = \Pr(M = 1|\mathrm{M} + \mathrm{F} = 2)$$

If we refer to Table 1 in the main paper, then $Q_1 = \frac{2\exp(\alpha_1)}{1+2\exp(\alpha_1)}$ and $Q_2 = \frac{2\exp(\alpha_2)}{1+2\exp(\alpha_2)}$. Under the null of parental allelic exchangeability, we expect:

$$\Pr(M = 1|\mathrm{M} + \mathrm{F} = 1) = 2\Pr(M = 0|\mathrm{M} + \mathrm{F} = 1)$$
$$\Pr(M = 1|\mathrm{M} + \mathrm{F} = 2) = 2\Pr(M = 2|\mathrm{M} + \mathrm{F} = 2)$$

Therefore, we are interested in the following test:

$$H_0: Q_1 = Q_2 = 2/3$$
$$H_A: Q_1 \neq 2/3 \text{ or } Q_2 \neq 2/3$$

Define:

- n_1 = the number of parents where $M+F=1$
- x_{01} = the number of parents where $M+F=1$ and $M = 0$
- x_{10} = the number of parents where $M+F=1$ and $M = 1$
- n_2 = the number of parents where $M+F=2$
- x_{20} = the number of parents where $M+F=2$ and $M=2$
- x_{11} = the number of parents where $M+F=2$ and $M=1$

Then we have the following binomial model:

$$\begin{aligned} p(x_{10}, x_{11}|n_1, n_2, Q_1, Q_2) &= \binom{n_1}{x_{10}}(Q_1)^{x_{10}}(1-Q_1)^{n_1-x_{10}}\binom{n_2}{x_{11}}(Q_2)^{x_{11}}(1-Q_2)^{n_2-x_{11}} \\ &= \binom{n_1}{x_{10}}(Q_1)^{x_{10}}(1-Q_1)^{x_{01}}\binom{n_2}{x_{11}}(Q_2)^{x_{11}}(1-Q_2)^{x_{20}} \end{aligned}$$

We differentiate the log likelihood:

$$\ell \sim x_{10}\log(Q_1) + x_{01}\log(1-Q_1) + x_{11}\log(Q_2) + x_{20}\log(1-Q_2)$$

The maximum likelihood estimates are $Q_1$and$Q_2$are:

$$\hat{Q}_1 = \frac{x_{10}}{x_{10} + x_{01}} = \frac{x_{10}}{n_1}$$

$$\hat{Q}_2 = \frac{x_{11}}{x_{11} + x_{20}} = \frac{x_{11}}{n_2}$$

The likelihood ratio test statistic (*LRTS*) is then as follows:

$$\begin{aligned}
LRTS &= -2\left(\ell\left(Q_1 = Q_2 = \frac{2}{3}\right) - \ell\left(Q_1 = \hat{Q}_1, Q_2 = \hat{Q}_2\right)\right) \\
&= -2\left(x_{10}\log\left(\frac{2}{3}\right) + x_{01}\log\left(\frac{1}{3}\right) + x_{11}\log\left(\frac{2}{3}\right) + x_{20}\log\left(\frac{1}{3}\right) - x_{10}\log\left(\hat{Q}_1\right)\right. \\
&\qquad \left. - x_{01}\log\left(1 - \hat{Q}_1\right) - x_{11}\log\left(\hat{Q}_2\right) - x_{20}\log\left(1 - \hat{Q}_2\right)\right) \\
&= -2\left(x_{10}\log\left(\frac{2}{3\hat{Q}_1}\right) + x_{01}\log\left(\frac{1}{3\left(1 - \hat{Q}_1\right)}\right) + x_{11}\log\left(\frac{2}{3\hat{Q}_2}\right)\right. \\
&\qquad \left. + x_{20}\log\left(\frac{1}{3\left(1 - \hat{Q}_2\right)}\right)\right) \\
&= -2\left(x_{10}\log\left(\frac{2n_1}{3x_{10}}\right) + x_{01}\log\left(\frac{n_1}{3x_{01}}\right) + x_{11}\log\left(\frac{2n_2}{3x_{11}}\right) + x_{20}\log\left(\frac{n_2}{3x_{20}}\right)\right)
\end{aligned}$$

The $LRTS$ is distributed chi-squared with 2 degrees of freedom under parental allelic exchangeability.

S.2: Closed form solutions for SSX-LRT

As in the main paper, we define *M*, *F*, and *C* as the number of variant alleles carried by the mother, father and child and we define the relative risk of being affected (aff), conditional on mating type (*M*, *F*) to control for population stratification, as:

$$R_B = \Pr(\text{aff}|\text{boy}, C = 1)\,/\Pr(\text{aff}|\text{boy}, C = 0)$$

$$R_{G1} = \Pr(\text{aff}|\text{girl}, C = 1)\,/\Pr(\text{aff}|\text{girl}, C = 0)$$

$$R_{G2} = R_{G1} * \Pr(\text{aff}|\text{girl}, C = 2)\,/\Pr(\text{aff}|\text{girl}, C = 1)$$

S.2.1 Triads with affected sons

We are interested in the following hypothesis test:

$$H_0: R_B = 1$$
$$H_A: R_B \neq 1$$

Define:

- n = the number of triads with a heterozygous mother (M=1)
- x = the number of triads with a heterozygous mother and an affected son with the variant allele (M=1, C=1)

Referring to Table 2 in the main paper, we have the following binomial model:

$$p(x|n, R_B) = \binom{n}{x}\left(\frac{R_B}{1+R_B}\right)^x\left(\frac{1}{1+R_B}\right)^{n-x} \quad \text{(A1)}$$

To find the maximum likelihood estimate of R_B ($\hat{R}_B$), we differentiate the log likelihood function (ignoring the constant terms) that corresponds to equation A1, set it to 0 and solve for $\hat{R}_B$:

$$\ell \sim x\log(R_B) - n\log(1+R_B)$$

$$\frac{d\ell}{dR_B} = \frac{x}{R_B} - \frac{n}{1+R_B}$$

$$0 = \frac{x}{\hat{R}_B} - \frac{n}{1+\hat{R}_B}$$

$$= (1+\hat{R}_B)x - \hat{R}_B n$$

$$\hat{R}_B = \frac{x}{n-x}$$

The likelihood ratio test statistic ($LRTS$) is then as follows:

$$\begin{aligned} LRTS &= -2\left(\ell(R_B = 1) - \ell(R_B = \hat{R}_B)\right) \\ &= -2\left(x\log\left(\frac{1}{2}\right) + (n-x)\log\left(\frac{1}{2}\right) - x\log\left(\frac{\hat{R}_B}{1+\hat{R}_B}\right) - (n-x)\log\left(\frac{1}{1+\hat{R}_B}\right)\right) \\ &= -2\left(n\log\left(\frac{1}{2}\right) - x\log(\hat{R}_B) + n\log(1+\hat{R}_B)\right) \\ &= -2\left(n\log\left(\frac{n}{2(n-x)}\right) - x\log\left(\frac{x}{n-x}\right)\right) \end{aligned}$$

The $LRTS$ is distributed chi-squared with 1 degree of freedom under the null.

S.2.2 Triads with affected daughters

We are interested in the following hypothesis:

$$H_0: R_{G1} = R_{G2} = 1$$
$$H_A: R_{G1} \neq 1 \text{ or } R_{G2} \neq 1$$

Define:

- n_1 = the number of triads with M=1 and F=0
- x_1 = the number of triads with M=1, F=0 and $C = 1$
- n_2 = the number of triads with M=1 and F=1
- x_2 = the number of triads with M=1, F=1 and $C = 2$

Referring to Table 2 in the main paper, we have the following binomial model:

$$p(x_1, x_2 | n_1, n_2, R_{G1}, R_{G2}) = \binom{n_1}{x_1}\left(\frac{R_{G1}}{1+R_{G1}}\right)^{x_1}\left(\frac{1}{1+R_{G1}}\right)^{n_1-x_1}\binom{n_2}{x_2}\left(\frac{R_{G2}}{R_{G1}+R_{G2}}\right)^{x_2}\left(\frac{R_{G1}}{R_{G1}+R_{G2}}\right)^{n_2-x_2} \quad \text{(A2)}$$

To find the maximum likelihood estimate of $R_{G1}$and R_{G2} ($\hat{R}_{G1}$ and $\hat{R}_{G2}$), we differentiate the log likelihood function that corresponds to equation A2, set it to 0 and solve for $\hat{R}_{G1}$ and $\hat{R}_{G2}$:

$$\ell \sim (x_1 + n_2 - x_2)\log(R_{G1}) - n_1 \log(1 + R_{G1}) + (x_2)\log(R_{G2}) - n_2 \log(R_{G1} + R_{G2})$$

To solve for $\hat{R}_{G2}$:

$$\frac{d\ell}{dR_{G2}} = \frac{x_2}{R_{G2}} - \frac{n_2}{R_{G1} + R_{G2}}$$

$$0 = \frac{x_2}{R_{G2}} - \frac{n_2}{R_{G1} + R_{G2}}$$

$$= (R_{G1} + R_{G2})(x_2) - R_{G2}(n_2)$$

$$\hat{R}_{G2} = \frac{R_{G1}(x_2)}{n_2 - x_2}$$

To solve for $\hat{R}_{G1}$:

$$\frac{d\ell}{dR_{G1}} = \frac{x_1 + n_2 - x_2}{R_{G1}} - \frac{n_1}{1 + R_{G1}} - \frac{n_2}{R_1 + \hat{R}_{G2}}$$

$$= \frac{x_1 + n_2 - x_2}{R_{G1}} - \frac{n_1}{1 + R_{G1}} - \frac{n_2}{R_{G1}\left(1 + \frac{x_2}{n_2 - x_2}\right)}$$

$$= \frac{x_1 + n_2 - x_2}{R_{G1}} - \frac{n_1}{1 + R_{G1}} - \frac{n_2 - x_2}{R_{G1}}$$

$$= \frac{x_1}{R_{G1}} - \frac{n_1}{1 + R_{G1}}$$

$$0 = \left(1 + \hat{R}_{G1}\right)x_1 - \hat{R}_{G1}n_1$$

$$\hat{R}_{G1} = \frac{x_1}{n_1 - x_1}$$

So, given $\hat{R}_{G1}$:

$$\hat{R}_{G2} = \frac{x_1 x_2}{(n_1 - x_1)(n_2 - x_2)}$$

The likelihood ratio test statistic (to be compared to a 2 DF chi-squared) is then as follows:

$$\begin{aligned}
LRTS &= -2\left(\ell(R_{G1} = R_{G2} = 1) - \ell\left(R_{G1} = \hat{R}_{G1}, R_{G2} = \hat{R}_{G2}\right)\right) \\
&= -2\Bigg(x_1 \log\left(\frac{1}{2}\right) + (n_1 - x_1)\log\left(\frac{1}{2}\right) + x_2 \log\left(\frac{1}{2}\right) + (n_2 - x_2)\log\left(\frac{1}{2}\right) \\
&\qquad - x_1 \log\left(\frac{\hat{R}_{G1}}{1 + \hat{R}_{G1}}\right) - (n_1 - x_1)\log\left(\frac{1}{1 + \hat{R}_{G1}}\right) - x_2 \log\left(\frac{\hat{R}_{G2}}{\hat{R}_{G1} + \hat{R}_{G2}}\right) \\
&\qquad - (n_2 - x_2)\log\left(\frac{\hat{R}_{G1}}{\hat{R}_{G1} + \hat{R}_{G2}}\right)\Bigg) \\
&= -2\left(n_1 \log\left(\frac{1 + \hat{R}_{G1}}{2}\right) + n_2 \log\left(\frac{\hat{R}_{G1} + \hat{R}_{G2}}{2\hat{R}_{G1}}\right) - x_1 \log\left(\hat{R}_{G1}\right) - x_2 \log\left(\frac{\hat{R}_{G2}}{\hat{R}_{G1}}\right)\right) \\
&= -2\Bigg(n_1 \log\left(\frac{n_1}{2(n_1 - x_1)}\right) + n_2 \log\left(\frac{n_2}{2(n_2 - x_2)}\right) - x_1 \log\left(\frac{x_1}{n_1 - x_1}\right) \\
&\qquad - x_2 \log\left(\frac{x_2}{n_2 - x_2}\right)\Bigg)
\end{aligned}$$

We can also consider a **log-additive** model such that:

$$H_0: R_{G1} = R_{G2} = 1$$
$$H_A: R_{G1}^2 = R_{G2} \neq 1$$

Then we have the following binomial model:

$$\begin{aligned}
&p(x_1, x_2 | n_1, n_2, R_1, R_2) \\
&\quad = \binom{n_1}{x_1}\left(\frac{R_{G1}}{1 + R_{G1}}\right)^{x_1}\left(\frac{1}{1 + R_{G1}}\right)^{n_1 - x_1}\binom{n_2}{x_2}\left(\frac{R_{G2}}{R_{G1} + R_{G2}}\right)^{x_2}\left(\frac{R_{G1}}{R_{G1} + R_{G2}}\right)^{n_2 - x_2}
\end{aligned}$$

$$= \binom{n_1}{x_1}\left(\frac{R_{G1}}{1+R_{G1}}\right)^{x_1}\left(\frac{1}{1+R_{G1}}\right)^{n_1-x_1}\binom{n_2}{x_2}\left(\frac{R_{G1}^2}{R_{G1}+R_{G1}^2}\right)^{x_2}\left(\frac{R_{G1}}{R_{G1}+R_{G1}^2}\right)^{n_2-x_2}$$
$$= \binom{n_1}{x_1}\left(\frac{R_{G1}}{1+R_{G1}}\right)^{x_1}\left(\frac{1}{1+R_{G1}}\right)^{n_1-x_1}\binom{n_2}{x_2}\left(\frac{R_{G1}}{1+R_{G1}}\right)^{x_2}\left(\frac{1}{1+R_{G1}}\right)^{n_2-x_2}$$
$$= \binom{n_1}{x_1}\binom{n_2}{x_2}\left(\frac{R_{G1}}{1+R_{G1}}\right)^{x_1+x_2}\left(\frac{1}{1+R_{G1}}\right)^{n_1+n_2-x_1-x_2} \quad \text{(A3)}$$

To find the maximum likelihood estimate of $R_{G1}$and R_{G2} ($\hat{R}_{G1}$ and $\hat{R}_{G2}$), we differentiate the log likelihood function that corresponds to equation A3, set it to 0 and solve for $\hat{R}_{G1}$ and $\hat{R}_{G2}$:

$$\ell \sim (x_1+x_2)\log(R_{G1}) - (n_1+n_2)\log(1+R_{G1})$$

$$\frac{d\ell}{dR_{G1}} = \frac{x_1+x_2}{R_{G1}} - \frac{n_1+n_2}{1+R_{G1}}$$

$$0 = \frac{x_1+x_2}{\hat{R}_{G1}} - \frac{n_1+n_2}{1+\hat{R}_{G1}}$$

$$\hat{R}_{G1} = \frac{x_1+x_2}{n_1+n_2-x_1-x_2}$$
$$\hat{R}_{G2} = \hat{R}_{G1}^2$$

The likelihood ratio test statistic (to be compared to a 1 DF chi-squared) is then as follows:

$$LRTS = -2\left(\ell(R_1 = R_2 = 1) - \ell\left(R_1 = \hat{R}_{G1}, R_2 = \hat{R}_{G1}^2\right)\right)$$
$$= -2\left((x_1+\mathrm{x}_2)\log\left(\frac{1}{2}\right) + (n_1+n_2-x_1-x_2)\log\left(\frac{1}{2}\right)\right.$$
$$\left. - (x_1+\mathrm{x}_2)\log\left(\frac{\hat{R}_{G1}}{1+\hat{R}_{G1}}\right) - (n_1+n_2-x_1-x_2)\log\left(\frac{1}{1+\hat{R}_{G1}}\right)\right)$$
$$= -2\left((n_1+\mathrm{n}_2)\log\left(\frac{1+\hat{R}_{G1}}{2}\right) - (x_1+x2)\log\left(\hat{R}_{G1}\right)\right)$$
$$= -2\left((n_1+\mathrm{n}_2)\log\left(\frac{n_1+n_2}{2(n_1+n_2-x_1-x_2)}\right)\right.$$
$$\left. - (x_1+x_2)\log\left(\frac{x_1+x_2}{n_1+n_2-x_1-x_2}\right)\right)$$

Similarly, we can calculate the maximum likelihood estimates and likelihood ratio test statistic for a **dominant** model where:

$$H_0: R_{G1} = R_{G2} = 1$$
$$H_A: R_{G1} = R_{G2} \neq 1$$

The MLEs of $R_{G1}$and $R_{G2}$are:

$$\hat{R}_{G2} = \hat{R}_{G1} = \frac{x_1}{n_1 - x_1}$$

The likelihood ratio test statistic (to be compared to a 1 DF chi-squared) is:

$$LRTS = -2\left(n_1 \log\left(\frac{n_1}{2(n_1 - x_1)}\right) - x_1 \log\left(\frac{x_1}{n_1 - x_1}\right)\right)$$

We calculate the maximum likelihood estimates and likelihood ratio test statistic for a **recessive** model where:

$$H_0: R_{G1} = R_{G2} = 1$$
$$H_A: R_{G1} = 1, R_{G2} \neq 1$$

The MLE of R_{G2} is:

$$\hat{R}_{G2} = \frac{x_2}{n_2 - x_2}$$

The likelihood ratio test statistic (to be compared to a 1 DF chi-squared) is:

$$LRTS = -2\left(n_2 \log\left(\frac{n_2}{2(n_2 - x_2)}\right) - x_1 \log\left(\frac{x_2}{n_2 - x_2}\right)\right)$$

Note that families in which the father carries the variant allele are not information under a dominant model. And only families in which the father carries the variant allele are informative under a recessive model.

S.3: Closed form solutions for the parent-only analysis

As in the main paper, we define *M*, *F*, and *C* as the number of variant alleles carried by the mother, father and child and we define the relative risk of being affected (aff), conditional on mating type (*M*, *F*) to control for population stratification, as:

$$R_B = \Pr(\text{aff}|\text{boy}, C = 1) / \Pr(\text{aff}|\text{boy}, C = 0)$$

$$R_{G1} = \Pr(\text{aff}|\text{girl}, C = 1) / \Pr(\text{aff}|\text{girl}, C = 0)$$

$$R_{G2} = R_{G1} * \Pr(\text{aff}|\text{girl}, C = 2) / \Pr(\text{aff}|\text{girl}, C = 1)$$

S.3.1 Triads with affected sons

We are interested in the following hypothesis test:

$$H_0: R_B = 1$$
$$H_A: R_B \neq 1$$

Define:

- n_1 = the number of triads with M+F=1
- x_1 = the number of triads with M+F=1 and M=1 and F=0
- n_2 = the number of triads with M+F=2
- x_2 = the number of triads with M+F=2 and M=2 and F=0

More generally, we have the following model (see Table 3 in paper):

$$p(x_1, x_2 | n_1, n_2, s) = \binom{n_1}{x_1}\left(\frac{1+R_B}{2+R_B}\right)^{x_1}\left(\frac{1}{2+R_B}\right)^{n_1 - x_1}\binom{n_2}{x_2}\left(\frac{R_B}{1+2R_B}\right)^{x_2}\left(\frac{1+R_B}{1+2R_B}\right)^{n_2-x_2} \qquad \text{(A4)}$$

To find the maximum likelihood estimate of R_B ($\hat{R}_B$), we differentiate the log likelihood function (ignoring the constant terms) that corresponds to A4, set it to 0 and solve for $\hat{R}_B$:

$$\ell \sim x_1 \log(1+R_B) - n_1 \log(2+R_B) + x_2 \log(R_B) + (n_2 - x_2)\log(1+R_B) - n_2 \log(1+2R_B)$$

$$\frac{d\ell}{ds} = \frac{x_1 + n_2 - x_2}{1+R_B} - \frac{n_1}{2+R_B} + \frac{x_2}{R_B} - \frac{2n_2}{1+2R_B}$$

$$0 = \frac{x_1 + n_2 - x_2}{1+\hat{R}_B} - \frac{n_1}{2+\hat{R}_B} + \frac{x_2}{\hat{R}_B} - \frac{2n_2}{1+2\hat{R}_B}$$
$$= \left(2\hat{R}_B + 5\hat{R}_B^2 + 2\hat{R}_B^3\right)(x_1 + n_2 - x_2) - \left(\hat{R}_B + 3\hat{R}_B^2 + 2\hat{R}_B^3\right)(n_1) - \left(2 + 7\hat{R}_B + 7\hat{R}_B^2 + 2\hat{R}_B^3\right)(x_2) - \left(2\hat{R}_B + 3\hat{R}_B^2 + \hat{R}_B^3\right)(2n_2)$$

$$= a_0 + a_1\hat{R}_B + a_2\hat{R}_B^2 + \hat{R}_B^3$$

where:

$$a_0 = \frac{x_2}{x_1 - n_1}$$

$$a_1 = \frac{2x_1 + 5x_2 - n_1 - 2n_2}{2x_1 - 2n_1}$$

$$a_2 = \frac{5x_1 + 2x_2 - 3n_1 - n_2}{2x_1 - 2n_1}$$

We can use Cardano's folmula to solve for the cubic. This solution was published in the 1500's by Gerolamo Cardano in *Ars Magna* (Cardano, 1545;Cardano and Witmer, 1993)

We are interested in the positive root for $\hat{R}_B$:

$$Q = \frac{3a_1 - {a_2}^2}{9}$$

$$R = \frac{9a_2a_1 - 27a_0 - 2{a_2}^3}{54}$$

$$D = Q^3 + R^2$$
$$S = \sqrt[3]{R + \sqrt{D}}$$
$$T = \sqrt[3]{R - \sqrt{D}}$$

The three roots are:

$$\left(-\frac{1}{3}\right)a_2 + (S + T)$$
$$\left(-\frac{1}{3}\right)a_2 - \frac{1}{2}\ (S + \square) + \frac{1}{2}i\sqrt{3}(S - T)$$

$$\left(-\frac{1}{3}\right)a_2 - \frac{1}{2}\ (S + T) - \frac{1}{2}i\sqrt{3}(S - T)$$

Let $\hat{R}_B$ be the positive, real root (if D>0, the first root). An approach using trigonometry was later developed as well and can be used to avoid the imaginary number (Nickalls, 2006).

The likelihood ratio test statistic (to be compared to a 1 DF chi-squared) is as follows:

$$\begin{aligned}
LRTS &= -2\left(\ell(R_B = 1) - \ell\left(R_B = \hat{R}_B\right)\right) \\
&= -2\Bigg(x_1 \log(2) - n_1 \log(3) + x_2\log\left(\frac{1}{2}\right) - n_2\log\left(\frac{3}{2}\right) - x_1\log\left(1 + \hat{R}_B\right) \\
&\qquad + n_1\log\left(2 + \hat{R}_B\right) - x_2\log\left(\frac{\hat{R}_B}{1 + \hat{R}_B}\right) + n_2\log\left(\frac{1 + 2\hat{R}_B}{1 + \hat{R}_B}\right)\Bigg) \\
&= -2\Bigg(x_1 \log\left(\frac{2}{1 + \hat{R}_B}\right) + n_1 \log\left(\frac{2 + \hat{R}_B}{3}\right) - x_2\log\left(\frac{2\hat{R}_B}{1 + \hat{R}_B}\right) \\
&\qquad + n_2\log\left(\frac{2\left(1 + 2\hat{R}_B\right)}{3\left(1 + \hat{R}_B\right)}\right)\Bigg)
\end{aligned}$$

S.3.2 Triads with affected daughters

We are interested in the following hypothesis:

$$H_0: R_{G1} = R_{G2} = 1$$
$$H_A: R_{G1} \neq 1 \text{ or } R_{G2} \neq 1$$

Define:

- n_1 = the number of triads where $M+F=1$
- x_1 = the number of triads where $M+F=1$ and $M = 1$ and $F = 0$
- n_2 = the number of triads where $M+F=2$
- x_2 = the number of triads where $M+F=2$ and $M = 2$ and $F = 0$

More generally, we have the following model (see Table 3 in paper):

$$p(x_1, x_2 | n_1, n_2, R_{G1}, R_{G2})$$

$$= \binom{n_1}{x_1}\left(\frac{1 + R_{G1}}{1 + 2R_{G1}}\right)^{x_1}\left(\frac{R_{G1}}{1 + 2R_{G1}}\right)^{n_1 - x_1} \binom{n_2}{x_2}\left(\frac{R_{G1}}{2R_{G1} + R_{G2}}\right)^{x_2}\left(\frac{R_{G1} + R_{G2}}{2R_{G1} + R_{G2}}\right)^{n_2 - x_2} \quad \text{(A5)}$$

To find the maximum likelihood estimate of $R_{G1}$and R_{G2} ($\hat{R}_{G1}$ and $\hat{R}_{G2}$), we differentiate the log likelihood function that corresponds to equation A5, set it to 0 and solve for $\hat{R}_{G1}$ and $\hat{R}_{G2}$:

$$\ell \sim x_1 \log(1 + R_{G1}) + (x_2 + n_1 - x_1)\log(R_{G1}) - n_1 \log(1 + 2R_{G1}) + (n_2 - x_2)\log(R_{G1} + R_{G2}) - n_2 \log(2R_{G1} + R_{G2})$$

$$\frac{d\ell}{dR_{G2}} = \frac{n_2 - x_2}{R_{G1} + R_{G2}} - \frac{n_2}{2R_{G1} + R_{G2}}$$

$$0 = \frac{n_2 - x_2}{R_{G1} + \hat{R}_{G2}} - \frac{n_2}{2R_{G1} + \hat{R}_{G2}}$$

$$= \left(2R_{G1} + \hat{R}_{G2}\right)(n_2 - x_2) - \left(R_{G1} + \hat{R}_{G2}\right)(n_2)$$

$$= \hat{R}_{G2}(x_2) + R_{G1}(2x_2 - n_2)$$

$$\hat{R}_{G2} = R_{G1}\frac{n_2 - 2x_2}{x_2}$$

$$\frac{d\ell}{dR_{G1}} = \frac{x_1}{1+R_{G1}} + \frac{x_2+n_1-x_1}{R_{G1}} - \frac{2n_1}{1+2R_{G1}} + \frac{n_2-x_2}{R_{G1}+\hat{R}_{G2}} - \frac{2n_2}{2R_{G1}+\hat{R}_{G2}}$$

$$= \frac{x_1}{1+R_{G1}} + \frac{x_2+n_1-x_1}{R_{G1}} - \frac{2n_1}{1+2R_{G1}} + \frac{n_2-x_2}{R_{G1}\left(1+\frac{n_2-2x_2}{x_2}\right)} - \frac{2n_2}{R_{G1}\left(2+\frac{n_2-2x_2}{x_2}\right)}$$

$$= \frac{x1}{1+R_{G1}} + \frac{x_2+n_1-x_1}{R_{G1}} - \frac{2n_1}{1+2R_{G1}} + \frac{x_2}{R_{G1}} - \frac{2x_2}{R_{G1}}$$

$$= \frac{x_1}{1+R_{G1}} + \frac{n_1-x_1}{R_{G1}} - \frac{2n_1}{1+2R_{G1}}$$

$$0 = (R_{G1}+2R_{G1}^2)x_1 + (1+3R_{G1}+2R_{G1}^2)(n_1-x_1) - (R_{G1}+R_{G1}^2)2n_1$$

$$= R_{G1}(n_1-2x_1) + n_1 - x_1$$

$$\hat{R}_{G1} = \frac{x_1-n_1}{n_1-2x_1}$$

The likelihood ratio test statistic (to be compared to a 2 DF chi-squared) is:

$$LRTS = -2\left(l(R_{G1}=R_{G2}=1) - l\big(R_{G1}=\hat{R}_{G1}, R_{G2}=\hat{R}_{G2}\big)\right)$$

$$= -2\Bigg(x_1\log(2) - n_1\log(3) - x_2\log(2) + n_2\log\left(\frac{2}{3}\right) - x_1\log\left(\frac{1+\hat{R}_{G1}}{\hat{R}_{G1}}\right)$$

$$- n_1\log\left(\frac{\hat{R}_{G1}}{1+2\hat{R}_{G1}}\right) - x_2\log\left(\frac{\hat{R}_{G1}}{\hat{R}_{G1}+\hat{R}_{G2}}\right) - n_2\log\left(\frac{\hat{R}_{G1}+\hat{R}_{G2}}{2\hat{R}_{G1}+\hat{R}_{G2}}\right)\Bigg)$$

$$= -2\Bigg(x_1\log\left(\frac{2(n_1-x_1)}{x_1}\right) - n_1\log\left(\frac{3(n_1-x_1)}{n_1}\right) - x_2\log\left(\frac{2x_2}{n_2-x_2}\right)$$

$$+ n_2\log\left(\frac{2n_2}{3(n_2-x_2)}\right)\Bigg)$$

We can also consider a **log-additive** model such that:

$$H_0: R_{G1} = R_{G2} = 1$$
$$H_A: R_{G1}^2 = R_{G2} \neq 1$$

More generally, we have the following model:

$$p(x_1, x_2 | n_1, n_2, R_{G1}, R_{G2})$$
$$= \binom{n_1}{x_1}\left(\frac{1+R_{G1}}{1+2R_{G1}}\right)^{x_1}\left(\frac{R_{G1}}{1+2R_{G1}}\right)^{n_1-x_1}\binom{n_2}{x_2}\left(\frac{R_{G1}}{2R_{G1}+R_{G2}}\right)^{x_2}\left(\frac{R_{G1}+R_{G2}}{2R_{G1}+R_{G2}}\right)^{n_2-x_2}$$

$$= \binom{n_1}{x_1}\left(\frac{1+R_{G1}}{1+2R_{G1}}\right)^{x_1}\left(\frac{R_{G1}}{1+2R_{G1}}\right)^{n_1-x_1} \times \binom{n_2}{x_2}\left(\frac{R_{G1}}{2R_{G1}+R_{G1}^2}\right)^{x_2}\left(\frac{R_{G1}+R_{G1}^2}{2R_{G1}+R_{G1}^2}\right)^{n_2-x_2}$$

$$= \binom{n_1}{x_1}\left(\frac{1+R_{G1}}{1+2R_{G1}}\right)^{x_1}\left(\frac{R_{G1}}{1+2R_{G1}}\right)^{n_1-x_1}\binom{n_2}{x_2}\left(\frac{1}{2+R_{G1}}\right)^{x_2}\left(\frac{1+R_{G1}}{2+R_{G1}}\right)^{n_2-x_2} \quad \text{(A6)}$$

To find the maximum likelihood estimate of $R_{G1}$and R_{G2} ($\hat{R}_{G1}$ and $\hat{R}_{G2}$), we differentiate the log likelihood function that corresponds to equation A6, set it to 0 and solve for $\hat{R}_{G1}$ and $\hat{R}_{G2}$:

$$\ell \sim x_1\log(1+R_{G1}) + (n_1-x_1)\log(R_{G1}) - n_1\log(1+2R_{G1}) + (n_2-x_2)\log(1+R_{G1}) - n_2\log(2+R_{G1})$$

$$\frac{d\ell}{dR_{G1}} = \frac{x_1+n_2-x_2}{1+R_{G1}} - \frac{n_2}{2+R_{G1}} + \frac{n_1-x_1}{R_{G1}} - \frac{2n_1}{1+2R_{G1}}$$

$$0 = \frac{x_1+n_2-x_2}{1+\hat{R}_{G1}} - \frac{n_2}{2+\hat{R}_{G1}} + \frac{n_1-x_1}{\hat{R}_{G1}} - \frac{2n_1}{1+2\hat{R}_{G1}}$$

$$= \left(2\hat{R}_{G1}+5\hat{R}_{G1}^2+2\hat{R}_{G1}^3\right)(x_1+n_2-x_2) - \left(\hat{R}_{G1}+3\hat{R}_{G1}^2+2\hat{R}_{G1}^3\right)(n_2) - \left(2+7\hat{R}_{G1}+7\hat{R}_{G1}^2+2\hat{R}_{G1}^3\right)(n_1-x_1) - \left(2\hat{R}_{G1}+3\hat{R}_{G1}^2+\hat{R}_{G1}^3\right)(2n_1)$$

$$= a_0 + a_1\hat{R}_{G1} + a_2\hat{R}_{G1}^2 + \hat{R}_{G1}^3$$

where:

$$a_0 = \frac{x_1-n_1}{x_2}$$

$$a_1 = \frac{2x_2+5x_1-n_2-3n_1}{2x_2}$$

$$a_2 = \frac{5x_2+2x_1-2n_2-n_1}{2x_2}$$

See section **S.3.1** for how to solve for $\hat{R}_{G1}$, then $\hat{R}_{G2} = \hat{R}_{G1}^2$.

The likelihood ratio test statistic (to be compared to a 1 DF chi-squared) is:

$$
\begin{aligned}
LRTS &= -2\left(\ell(R_{G1} = R_{G2} = 1) - \ell\left(R_{G1} = \hat{R}_{G1}\,, R_{G2} = \hat{R}_{G1}^2\right)\right) \\
&= -2\left(x_1 \log(2) - n_1 \log(3) + x_2 \log\left(\frac{1}{2}\right) - n_2 \log\left(\frac{3}{2}\right) - x_1 \log\left(\frac{1+\hat{R}_{G1}}{\hat{R}_{G1}}\right)\right. \\
&\qquad \left. + n_1 \log\left(\frac{1+2\hat{R}_{G1}}{\hat{R}_{G1}}\right) - x_2 \log\left(\frac{1}{1+\hat{R}_{G1}}\right) + n_2 \log\left(\frac{2+\hat{R}_{G1}}{1+\hat{R}_{G1}}\right)\right) \\
&= -2\left(x_1 \log\left(\frac{2\hat{R}_{G1}}{1+\hat{R}_{G1}}\right) + n_1 \log\left(\frac{1+2\hat{R}_{G1}}{3\hat{R}_{G1}}\right) + x_2 \log\left(\frac{1+\hat{R}_{G1}}{2}\right)\right. \\
&\qquad \left. + n_2 \log\left(\frac{2\left(2+\hat{R}_{G1}\right)}{3\left(1+\hat{R}_{G1}\right)}\right)\right)
\end{aligned}
$$

Similarly for triads with affected daughters, we can calculate the maximum likelihood estimates and likelihood ratio test statistic for a **dominant** or **recessive** model (results not shown).

S.4: Closed form solutions for PIX-LRT

In this section we define a likelihood that involves both the transmission-based information and the parental information. As in the main paper, we define *M*, *F*, and *C* as the number of variant alleles carried by the mother, father and child and we define the relative risk of being affected (aff), conditional on mating type (*M*,*F*) to control for population stratification, as:

$$R_B = \Pr(\text{aff}|\text{boy}, C = 1)\,/\Pr(\text{aff}|\text{boy}, C = 0)$$

$$R_{G1} = \Pr(\text{aff}|\text{girl}, C = 1)\,/\Pr(\text{aff}|\text{girl}, C = 0)$$

$$R_{G2} = R_{G1} * \Pr(\text{aff}|\text{girl}, C = 2)\,/\Pr(\text{aff}|\text{girl}, C = 1)$$

S.4.1 Triads with affected sons

We are interested in the following hypothesis test:

$$H_0\colon R_B = 1$$
$$H_A\colon R_B \neq 1$$

Define:

- n_1 = the number of triads where M+F=1
- x_1 = the number of triads where M+F=1 and C=1

- n_2 = the number of triads where M+F=2
- x_2 = the number of triads where M+F=2 and C=1

We have the following model:

$$p(x_1, x_2 | n_1, n_2, R_B) = \binom{n_1}{x_1}\left(\frac{R_B}{2+R_B}\right)^{x_1}\left(\frac{2}{2+R_B}\right)^{n_1-x_1}\binom{n_2}{x_2}\left(\frac{2R_B}{1+2R_B}\right)^{x_2}\left(\frac{1}{1+2R_B}\right)^{n_2-x_2}$$

The likelihood and ML estimate are as follows:

$$\ell \sim x_1 \log(R_B) - n_1 \log(2+R_B) + x_2 \log(R_B) - n_2 \log(1+2R_B)$$

$$\frac{d\ell}{dR_B} = \frac{x_1+x_2}{R_B} - \frac{n_1}{2+R_B} - \frac{2n_2}{1+2R_B}$$

$$0 = \left(2 + 5\hat{R}_B + 2\hat{R}_B^2\right)(x_1+x_2) - \left(\hat{R}_B + 2\hat{R}_B^2\right)(n_1) - \left(2\hat{R}_B + \hat{R}_B^2\right)(2n_2)$$

$$= \hat{R}_B^2\left(2(x_1 + x_2 - n_1 - n_2)\right) + \hat{R}_B(5x_1 + 5x_2 - n_1 - 4n_2) + 2(x_1+x_2)$$

If:
$a = 2(x_1 + x_2 - n_1 - n_2)$
$b = 5x_1 + 5x_2 - n_1 - 4n_2$
$c = 2(x_1 + x_2)$

Then $\hat{R}_B = \frac{\left(-b - \sqrt{b^2-4ac}\right)}{2a}$ and the LRT statistic is:

The likelihood ratio test statistic (to be compared to a 1 DF chi-squared) is:

$$\begin{aligned} LRTS &= -2\left(\ell(R_B = 1) - \ell\left(R_B = \hat{R}_B\right)\right) \\ &= -2\Big(x_1 \log(1) - n_1 \log(3) + x_2 \log(1) - n_2 \log(3) - x_1 \log\left(\hat{R}_B\right) \\ &\qquad + n_1 \log\left(2+\hat{R}_B\right) + x_2 \log\left(\hat{R}_B\right) + n_2 \log\left(1+2\hat{R}_B\right)\Big) \\ &= -2\left(x_1 \log\left(\frac{1}{\hat{R}_B}\right) + n_1 \log\left(\frac{2+\hat{R}_B}{3}\right) + x_2 \log\left(\frac{1}{\hat{R}_B}\right) + n_2 \log\left(\frac{1+\hat{R}_B}{3}\right)\right) \end{aligned}$$

S.4.2 Triads with affected daughters

Define:

- n_1 = the number of triads where M+F = 1
- x_1 = the number of triads where M+F = 1 and C = 1
- n_2 = the number of triads where M+F = 2

- x_2 = the number of triads where M+F = 2 and C = 2

We are interested in testing the following hypothesis:

$$H_0: R_{G1} = R_{G2} = 1$$
$$H_A: R_{G1} \neq 1, R_{G2} \neq 1$$

We have the following model:

$$p(x_1 , x_2 | n_1, n_2, R_{G1}, R_{G2})$$
$$= \binom{n_1}{x_1}\left(\frac{2R_{G1}}{1+2R_{G1}}\right)^{x_1}\left(\frac{1}{1+2RG_1}\right)^{n_1-x_1} \binom{n_2}{x_2}\left(\frac{R_{G2}}{2R_{G1}+R_{G2}}\right)^{x_2}\left(\frac{2R_{G1}}{2R_{G1}+R_{G2}}\right)^{n_2-x_2}$$

The likelihood and ML estimates for R_1 and R_2 are as follows:

$$\ell \sim (x_1 + n_2 - x_2)\log(R_{G1}) - n_1 \log(1 + 2R_{G1}) + x_2 \log(R_{G2}) - n_2 \log(2R_{G1} + R_{G2})$$

$$\frac{d\ell}{dR_{G2}} = \frac{x_2}{R_{G2}} - \frac{n_2}{2R_{G1} + R_{G2}}$$

$$0 = x_2\left(2R_{G1} + \hat{R}_{G2}\right) - n_2\left(\hat{R}_{G2}\right)$$

$$\hat{R}_{G2} = \frac{2R_{G1}x_2}{n_2 - x_2}$$

$$\frac{d\ell}{dR_{G1}} = \frac{x_1 + n_2 - x_2}{R_{G1}} - \frac{2n_1}{1 + 2R_{G1}} - \frac{2n_2}{2R_{G1} + R_{G2}}$$

$$0 = \frac{x_1 + n_2 - x_2}{\hat{R}_{G1}} - \frac{2n_1}{1 + 2\hat{R}_{G1}} - \frac{2n_2}{2\hat{R}_{G1} + \hat{R}_{G2}}$$
$$= \frac{x_1 + n_2 - x_2}{\hat{R}_{G1}} - \frac{2n_1}{1 + 2\hat{R}_{G1}} - \frac{2n_2}{2\hat{R}_{G1}\left(\frac{n_2}{n_2 - x_2}\right)}$$
$$= \left(1 + 2\hat{R}_{G1}\right)x_1 - 2\hat{R}_{G1}n_1$$

$$\hat{R}_{G1} = \frac{x_1}{2(n_1 - x_1)}$$

The likelihood ratio test statistic (to be compared to a 1 DF chi-squared) is:

$$LRTS = -2\left(\ell(R_{G1} = R_{G2} = 1) - \ell\left(R_{G1} = \hat{R}_{G1}, R_{G2} = \hat{R}_{G2}\right)\right)$$

$$= -2\Big(x_1 \log(1) - n_1 \log(3) + x_2 \log(1) - n_2 \log(3) - x_1 \log(\hat{R}_{G1}) + n_1 \log(1 + 2\hat{R}_{G1}) - x_2 \log\left(\frac{\hat{R}_{G2}}{\hat{R}_{G1}}\right) + n_2 \log\left(\frac{2\hat{R}_{G1} + \hat{R}_{G2}}{\hat{R}_{G1}}\right)\Big)$$

$$= -2\left(x_1 \log\left(\frac{2(n_1 - x_1)}{x_1}\right) + n_1 \log\left(\frac{n_1}{3(n_1 - x_1)}\right) + x_2 \log\left(\frac{n_2 - x_2}{2x_2}\right) + n_2 \log\left(\frac{2n_2}{3(n_2 - x_2)}\right)\right)$$

We can also consider a **log-additive** model such that:

$$H_0: R_{G1} = R_{G2} = 1$$
$$H_A: R_{G1}^2 = R_{G2} \neq 1$$

Our model is as follows:

$$p(x_1, x_2 | n_1, n_2, R_1, R_2)$$
$$= \binom{n_1}{x_1}\left(\frac{2R_{G1}}{1 + 2R_{G1}}\right)^{x_1}\left(\frac{1}{1 + 2R_{G1}}\right)^{n_1 - x_1}\binom{n_2}{x_2}\left(\frac{R_{G2}}{2R_{G1} + R_{G2}}\right)^{x_2}\left(\frac{2R_{G1}}{2R_{G1} + R_{G2}}\right)^{n_2 - x_2}$$
$$= \binom{n_1}{x_1}\left(\frac{2R_{G1}}{1 + 2R_{G1}}\right)^{x_1}\left(\frac{1}{1 + 2R_{G1}}\right)^{n_1 - x_1}\binom{n_2}{x_2}\left(\frac{R_1^2}{2R_1 + R_1^2}\right)^{x_2}\left(\frac{2R_1}{2R_1 + R_1^2}\right)^{n_2 - x_2}$$
$$= \binom{n_1}{x_1}\left(\frac{2R_1}{1 + 2R_1}\right)^{x_1}\left(\frac{1}{1 + 2R_1}\right)^{n_1 - x_1}\binom{n_2}{x_2}\left(\frac{R_{G1}}{2 + R_{G1}}\right)^{x_2}\left(\frac{2}{2 + R_{G1}}\right)^{n_2 - x_2}$$

The likelihood and ML estimates for R_1 and R_2 are as follows:

$$\ell \sim (x_1) \log(R_{G1}) - n_1 \log(1 + 2R_{G1}) + x_2 \log(R_{G1}) - n_2 \log(2 + R_{G1})$$

$$\frac{d\ell}{dR_{G1}} = \frac{x_1 + x_2}{R_{G1}} - \frac{2n_1}{1 + 2R_{G1}} + \frac{n_2}{2 + R_{G1}}$$

$$0 = \frac{x_1 + x_2}{\hat{R}_{G1}} - \frac{2n_1}{1 + 2\hat{R}_{G1}} + \frac{n_2}{2 + \hat{R}_{G1}}$$

$$= (2 + 5\hat{R}_{G1} + 2\hat{R}_{G1}^2)(x_1 + x_2) - (2\hat{R}_{G1} + \hat{R}_{G1}^2)(2n_1) - (\hat{R}_{G1} + 2\hat{R}_{G1}^2)(n_2)$$

$$= a\hat{R}_{G1}^2 + b\hat{R}_{G1} + c$$

Where:

$a = 2(x_1 + x_2 - n_1 - n_2)$
$b = 5x_2 + 5x_1 - 4n_1 - n_2$
$c = 2(x_1 + x_2)$

So $\hat{R}_{G1} = \frac{-b-\sqrt{b^2-4ac}}{2a}$ and $\hat{R}_{G2} = \hat{R}_{G1}^2$. The LRT statistic (to be compared to a 1 DF chi-squared) is:

$$\begin{aligned} LRTS &= -2\left(\ell(R_{G1} = R_{G2} = 1) - \ell\left(R_{G1} = \hat{R}_{G1}, R_{G2} = \hat{R}_{G1}^2\right)\right) \\ &= -2\big(x_1 \log(1) - n_1 \log(3) + x_2 \log(1) - n_2 \log(3) - x_1 \log\big(\hat{R}_{G1}\big) \\ &\qquad + n_1 \log\big(1 + 2\hat{R}_{G1}\big) - x_2 \log\big(\hat{R}_{G1}\big) + n_2 \log\big(2 + \hat{R}_{G1}\big)\big) \\ &= -2\left(x_1 \log\left(\frac{1}{\hat{R}_{G1}}\right) + n_1 \log\left(\frac{1 + 2\hat{R}_{G1}}{3}\right) + x_2 \log\left(\frac{1}{\hat{R}_{G1}}\right) + n_2 \log\left(\frac{2 + \hat{R}_{G1}}{3}\right)\right) \end{aligned}$$

Similarly for triads with affected daughters, we can calculate the maximum likelihood estimates and likelihood ratio test statistic for a **dominant** or **recessive** model (results not shown).

Supplement References

Cardano, G. (1545). *Hieronymi Cardani, praestantissimi mathematici, philosophi, ac medici, artis magnae, siue, De regulis algebraicis lib. unus : qui & totius operis de arithmetica, quod opus perfectum inscripsit, est in ordine decimus.* Norimbergae: Per Ioh. Petreium excusum.

Cardano, G., and Witmer, T.R. (1993). *Ars magna, or, The rules of algebra.* New York: Dover.

Nickalls, R.W.D. (2006). Viète, Descartes and the cubic equation. *Mathematical Gazette* 90.
